# Reductions in biomarkers of exposure to selected harmful and potentially harmful constituents following exclusive and partial switching from combustible cigarettes to *my*blu^™^ electronic nicotine delivery systems (ENDS)

**DOI:** 10.1007/s11739-021-02813-w

**Published:** 2021-08-26

**Authors:** Paul Morris, Simon McDermott, Fiona Chapman, Thomas Verron, Xavier Cahours, Matthew Stevenson, Joseph Thompson, Nveed Chaudhary, Grant O’Connell

**Affiliations:** 1 Nerudia Ltd—an Imperial Brands PLC Company, Wellington House, Physics Road, Speke, Liverpool, L24 9HP UK; 2 Imperial Brands PLC, 121 Winterstoke Road, Bristol, BS3 2LL UK

**Keywords:** Tobacco harm reduction, e-Cigarettes, Electronic nicotine delivery systems, Biomarkers of exposure, Smoking, Cigarette

## Abstract

Electronic nicotine delivery systems (ENDS) offer adult combustible cigarette smokers an alternative, potentially reduced harm, mode of nicotine delivery, attributed to fewer and reduced levels of harmful and potentially harmful constituents (HPHCs) in their aerosols compared to cigarette smoke. These two identical, randomised, open label, two-part studies aimed to compare levels of 15 biomarkers of exposure (BoE) to selected HPHCs associated with tobacco smoking in healthy US adult smoker subjects (*n* = 72). Following 9 days of exclusive use of a range of allocated *my*blu^™^ ENDS variants, subjects’ levels of 14 non-nicotine BoE were substantially reduced compared to baseline values (combustible cigarette use), in the range of 46–97%. BoE reductions were sustained in subjects who continued *my*blu use exclusively (*n* = 25) for a further 5 days, and returned to near baseline levels in subjects who returned to exclusive combustible cigarette use (*n* = 21). Dual users (*n* = 24) demonstrated reductions in BoE to a lesser extent than with exclusive *my*blu use. Measured nicotine equivalents did not significantly change throughout the study. These data suggest exclusive use of ENDS provides adult smokers seeking an alternative to combustible cigarettes with substantial reductions in HPHC exposures whilst achieving satisfying levels of nicotine delivery. Dual use involving substitution of cigarettes may also provide some of this advantage, but to lesser extent. Overall, the data contribute to the weight of evidence that ENDS are an important tool in tobacco harm reduction for adult smokers unwilling to or uninterested in quitting smoking. Study 1: NCT 04430634, study 2: NCT 04429932, clinicaltrials.gov (10-06-2020).

**Supplementary Information:**

The online version contains supplementary material available at 10.1007/s11739-021-02813-w.

## Introduction

Tobacco harm reduction (THR) involves providing a means by which adult combustible cigarette smokers, who are uninterested or unwilling to quit smoking, can achieve satisfactory nicotine consumption but with exposure to fewer, and substantially reduced levels of, toxicants associated with burning tobacco [1, 2]. This is an increasingly important concept and electronic nicotine delivery systems (ENDS) are one such category which may contribute to THR; a growing body of evidence within the scientific literature supports this, along with a number of regulators and public health bodies [1–4]. Recently, a comprehensive Cochrane Review found ENDS are an effective tool in enabling smoking cessation in adult smokers, even among those who do not intend to quit smoking, with no serious unwanted effects or harm with up to 2 years of usage [5]. Clinical data suggest that adult smokers who transition to use of ENDS generally experience improvements in their pulmonary and cardiovascular health [6, 7] and there is increasing evidence that the reduced harm potential of ENDS is linked to fewer and/or reduced levels of harmful and potentially harmful constituents (HPHCs) present in their aerosols, compared to smoke generated from cigarette combustion [8–12]. Rudd et al. [8] found that differences in toxicological outcomes in vitro directly correlated with HPHC exposure levels: reduced levels and fewer numbers of HPHCs within ENDS aerosol resulted in substantially reduced cytotoxicity, and negative outcomes for both genotoxicity and mutagenicity, unlike exposure to cigarette smoke. Similar reductions in toxicity of ENDS aerosols when compared to cigarette smoke samples have been recorded in a number of other studies, for example: [13–16].

Further to observations on smoke/aerosol compositions within laboratory settings, a number of clinical studies have also indicated substantially reduced biomarkers of exposure BoE to tobacco smoke HPHCs in adult smokers following exclusive use of ENDS [17–20]. For example, when adult smokers switched to a period of exclusive use of an ENDS device, they experienced substantial reductions in exposure to HPHCs, and in a short amount of time, to levels not dissimilar to those found in adult smokers who had switched to medically licensed nicotine replacement therapies (NRT) or ‘cold turkey’ cessation [21–23]. In general, previous studies have investigated the effects of older generation ENDS, however, there are fewer studies analysing the BoE levels associated with newer generation ENDS use; these newer generation products are now a popular alternative for combustible cigarettes used by adult smokers to reduce or replace smoking while still consuming nicotine [24, 25]. Newer generation products commonly take the form of a pod-based system, where a battery-powered segment is combined with a pre-filled ‘pod’ containing the e-liquid [8, 26].

Nicotine can be present in a freebase or nicotine salt form within the e-liquid to satisfy the preference of the adult smoker [27]. Nicotine salt, produced by the reaction of nicotine and a suitable acid, provides improved nicotine delivery to the adult smoker’s lung than the more volatile freebase variant and, therefore, results in greater pulmonary absorption, increased blood nicotine levels, and adult smoker satisfaction [28]. A range of flavour/nicotine options has been evidenced to play a role in ‘off-ramping’ of adult smokers from combustible cigarettes to potentially less harmful forms of nicotine consumption, including ENDS use, by increasing the acceptability and satisfaction of such products based on personal preference [5, 29, 30]. In fact, an important and common part of this off-ramping process is a period of ‘dual-use’ transition, where adult smokers will continue to use combustible cigarettes but will combine this with ENDS use as they familiarise themselves with these products and gradually reduce the number of combustible cigarettes smoked per day [31]. It has been demonstrated that dual-users are more motivated to quit smoking and are less dependent on combustible cigarette consumption [32]. In fact, there is also some evidence that there is an increased likelihood of dual-users going on to replace cigarette smoking entirely compared to those adult smokers who do not dual use [33]. Additionally, it has been found that adult smokers who become dual-users, and subsequently reduce their combustible cigarette use, experience reduced exposures to combustible tobacco BoE [22, 34]. Therefore, a dual-use arm was included in this study to further investigate and quantify the effects of dual use of newer generation ENDS and cigarettes on tobacco smoking BoE.

This study aimed to assess the changes from baseline (Day 1, cigarette smoking) levels of 15 tobacco-smoking related BoE following a 9-day exclusive use period of *my*blu^™^ ENDS products. Given some recent societal concerns around the potential additional toxicological burden of e-liquid flavourings, different nicotine strengths and the use of nicotine salts [35, 36], we sought to include a range of products (currently available on the US market) to represent these formulaic differences within this study. The second, subsequent, part of this study aimed to assess (in randomised groups) (i) if any changes in BoE levels (compared to baseline) following exclusive *my*blu use for 9 days were maintained up to 14 days, (ii) the effects on BoE levels (against baseline) following participants’ switching back to cigarette smoking between study Days 9 and 14 and (iii) the effects of dual use of cigarettes and *my*blu ENDS on BoE (against baseline) following participants’ switching to this between days 9 and 14. We selected the 15 BoE [primary: carboxyhemoglobin (COHb; corresponding HPHC, carbon monoxide), 4-(methylnitrosamino)-1-(3-pyridyl)-1-butanol (NNAL; 4-(methylnitrosamino)-1-(3-pyridyl)-1-butanone), 3-hydroxypropyl-mercapturic acid (3-HPMA; acrolein), S-phenyl-mercapturic acid (S-PMA; benzene); secondary: 2-cyanoethylmercapturic acid (CEMA; acrylonitrile), hydroxyethyl mercapturic acid (HEMA; ethylene oxide), 3-hydroxy-1-methylpropyl-mercapturic acid (3-HMPMA; crotonaldehyde), monohydroxybutenyl-mercapturic acid (MHBMA; 1,3-butadiene), o-toluidine (o-tol; toluidine), 1-aminonaphthalene (1-AN; naphthalene), 2-aminonaphthalene (2-AN; naphthalene), N-nitrosonornicotine (NNN; N-nitrosonornicotine), 1-hydroxypyrene (1-OHP; pyrene), 3-hydroxybenzo[a]pyrene (3-OH B[a]P; benzo[a]pyrene), nicotine equivalents (nicotine)] to represent major classes of compounds found within cigarette smoke, the majority of which have been reported by the US FDA to significantly contribute, or potentially contribute, to smoking-related disease risks [37]. This study complements existing pre-clinical toxicological data on the *my*blu ENDS test samples [8, 38] and acts to verify those findings within adult combustible cigarette smoking subjects.

## Methods

### Study participants

Study participants were recruited from areas surrounding the study sites (Celerion, Lincoln, NE; Frontage, Secaucus, NJ) using standard advertising methods and were compensated for their participation in the study. Seventy-two subjects completed the study and met the conditions for inclusion in the data analysis (out of a total of 79 recruited); the study population consisted of healthy US adult smokers aged 21–65 years, who reported smoking ten or more commercially available combustible cigarettes (menthol or non-menthol) per day (for a period of at least 12 months) and did not use any other nicotine-containing products within the 14 days prior to check-in (Day 2). Smoking > 10 cigarettes per day is a commonly used criterion which represents a typical/moderate level of product usage for regular adult smokers [39, 40]. As the study aimed to assess the potential of the test products to offer adult smokers an alternative mode of nicotine delivery with THR potential due to reduced exposure to HPHCs, exclusive smoker adults who were prepared to switch for the duration of the study but were otherwise not intending to alter their smoking habits were recruited for this study. The participants self-reported that they had not used other nicotine-containing products for 14 days prior to the study, and this criterion was applied to remove any confounding effects on measurements from such products’ use. However, ENDS or other nicotine product use prior to this 14 day period were not restricted. Smoking status was confirmed via urine cotinine (≥ 200 ng/mL) and exhaled carbon monoxide (> 10 ppm) levels [41].

Study exclusion criteria included a history or presence of clinically significant mental or physical health conditions; females who were pregnant or breastfeeding; high blood pressure (vital sign ranges used at screening were based on the clinic sites’ standard ranges: systolic blood pressure 90–150 mmHg, diastolic blood pressure 40–95 mmHg and heart rate 40–99 bpm); body mass index < 18 kg/m^2^ or > 40 kg/m^2^; acute illnesses (e.g., upper respiratory infection, viral infection) requiring treatment within 14 days prior to check-in; use of prescription smoking cessation treatments, anti-diabetic or insulin drugs or medications known to interact with Cytochrome P450 2A6; positive urine screen for alcohol or drugs of abuse; and self-reported puffers or mouth-hold smokers (i.e., smokers who draw smoke from a combustible cigarette into the mouth and throat but do not inhale).

The studies were carried out in accordance with: title 21 of the Code of Federal Regulations (21 CFR) parts 50, 56, 312; the US FDA electronic nicotine delivery systems guidance for industry [42] for biomarker endpoint assessment; International Council on Harmonisation (ICH) guidelines regarding Good Clinical Practice (E6 Consolidated Guidance, April 1996) [43]; the ethical principles set forth in the Declaration of Helsinki; all other relevant laws including those in relation to the protection of subjects data under the General Data Protection Regulation (GDPR); the approved study protocols. The study protocol for study 1 was reviewed and approved by Advarra Institutional Review Board (IRB) (Columbia, MD) and the study protocol for Study 2 was reviewed and approved by IntegReview IRB (Austin, TX). Any subsequent amendments were also approved by the same respective IRBs. Both the Advarra IRB and IntegReview IRB were constituted and operated in accordance with the principles and requirements described in the 21 CFR Part 56; both IRBs are compliant to ICH guidelines. All subjects provided informed consent. All prospective subjects had the study explained to them by the Investigator or their designee and were required to read, sign and date an Institutional Review Board (IRB) approved Informed Consent Form (ICF) prior to completion of screening or other study procedures. This consent form provided the subjects in non-technical terms with the purpose of the study, procedures to be carried out, and potential hazards. The subjects were assured that they may withdraw from the study at any time without jeopardising medical care related to or required as a result of study participation. Subjects were given a copy of their signed ICF. Study 1 (CA22749) was registered at www.clinicaltrials.gov with ID: NCT 04430634. Study 2 (CA22736) study was registered at www.clinicaltrials.gov with ID: NCT 04429932.

### Products

The ENDS test products used in this study were the commercially available *my*blu^™^ two-piece closed system comprised of a rechargeable 350 mAh battery and disposable pod containing an e-liquid. The e-liquid mixtures consisted of VG, PG, nicotine and a proprietary blend of flavours; pods contained 1.5 mL of e-liquid, equating to approximately 200 puffs under standardised machine puffing conditions [28]. Sixteen commercial disposable liquid pod variants were included in this study (Table 1). The pods also include the mouthpiece and heating element, and connect directly to, and are only compatible with, the *my*blu ENDS battery section. During use, the consumer inhaled through the mouthpiece and a sensor in the battery section detected the resulting change in air pressure, and in turn the heating element in the pod was activated, heating the e-liquid to an aerosol subsequently inhaled by the user. An example image of the product can be found in the supplementary information (Fig. 4).

**Table 1:** Details of the *my*blu ENDS product variants used in Studies 1 and 2

Product identifier	Product variant	Nicotine strength (w/v)	Nicotine form
Study 1			
1A	Intense tobacco (2.4%)	24 mg/mL	Nicotine salt
1B	Intense melon mint (3.6%)	36 mg/mL	Nicotine salt
1C	Intense fresh melon (2.5%)	25 mg/mL	Nicotine salt
1D	Intense tangerine cream (4.0%)	40 mg/mL	Nicotine salt
1E	Intense tobacco (3.6%)	36 mg/mL	Nicotine salt
1F	Intense melon mint (2.4%)	24 mg/mL	Nicotine salt
1G	Intense fresh melon (4.0%)	40 mg/mL	Nicotine salt
1H	Intense fresh mint (3.6%)	36 mg/mL	Nicotine salt
Study 2			
2A	Gold leaf (2.4%)	24 mg/mL	Freebase nicotine
2B	Polar mint (2.4%)	24 mg/mL	Freebase nicotine
2C	Cherry (2.4%)	24 mg/mL	Freebase nicotine
2D	Vanilla (2.4%)	24 mg/mL	Freebase nicotine
2E	Gold leaf (1.2%)	12 mg/mL	Freebase nicotine
2F	Polar mint (1.2%)	12 mg/mL	Freebase nicotine
2G	Menthol (2.4%)	24 mg/mL	Freebase nicotine
2H	Intense fresh mint (2.4%)	24 mg/mL	Nicotine salt

Products were assigned codes A–H for randomisation of product use during Part 1 of the study

The ENDS were charged and assembled for the participants and product information sheets provided. On each Study Day, fresh pods and a fully charged device were provided. All participants received training from clinic staff on how to operate their ENDS and to ensure compliance in the clinic, all participants used their products under the supervision of suitably qualified staff.

### Study design and procedure

The study was conducted under the oversight of Celerion in two US-based clinical research centres (Celerion, Lincoln, NE; Frontage, Secaucus, NJ). Two identical studies were split between the two locations to enable recruitment of the required numbers of participants (based on subject availability on volunteer databases at the time of the study) and according to clinical space available at the time of the study. Subjects were enrolled to participate in a randomised, open label, two-part study. All subjects participating in the study were expected to participate in both study parts, with Part 2 continuing immediately on from Part 1. Subjects were confined to the respective clinics for the full duration of the study. The objectives of the study were the measurement of change-from-baseline differences in combustible cigarette smoking-related BoE following 9 days of exclusive use of a variety of *my*blu ENDS products (Part 1), then to assess the differences in change from baseline following randomisation of participants to (I) exclusive use of allocated *my*blu ENDS products, (J) exclusive combustible cigarette smoking or (K) dual use of combustible cigarettes and *my*blu ENDS products between days 9 and 14 (Part 2). Part 1 of the study was over 9 days to align with data collection for a pharmacokinetic (PK) study (data not presented here); the 9 day period was also selected as an appropriate timeframe to observe reductions in BoE, as seen with previous studies over shorter time periods [17, 28]. The 5 day period selected for Part 2 of the study was deemed sufficient for the detection of changes in biomarkers [17] following switching of participants to their respective product use arms.

**Fig. 1:**
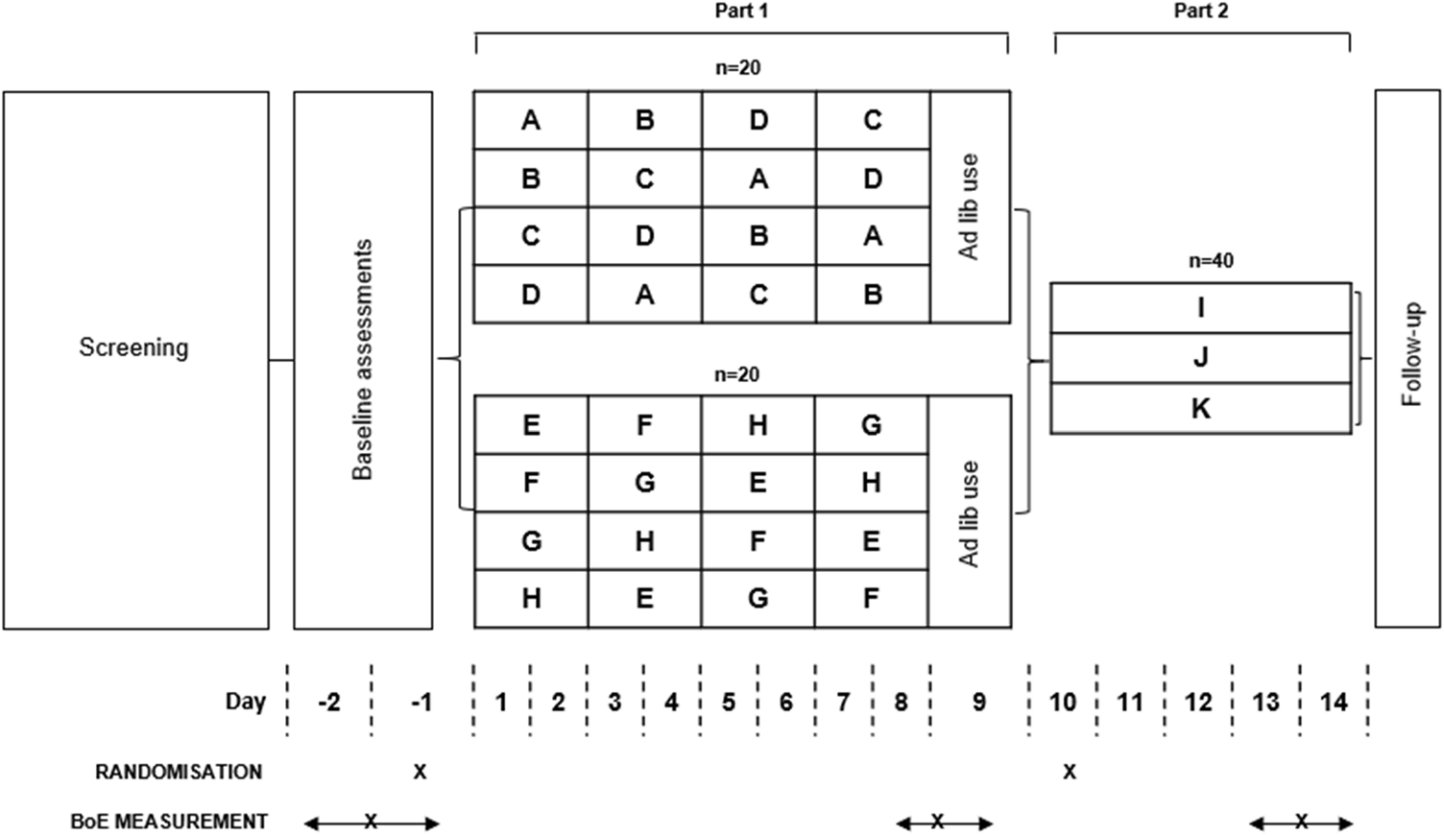
Overview of the study design. Following screening, participants checked-in to their respective clinical research unit on Day 2. On Days 2 through 1 (24 h) baseline BoE assessments were made followed by randomisation to the *my*blu ENDS product use sequences detailed, Days 1 through 9 (Part 1). Details of the products (A–H) for identical Studies 1 and 2 can be found in Table 1 (*n* = number of participants intended to participate in one Study). In Part 2 of the Studies, participants were randomised to one of three study arms (I, J or K, detailed in Table 2). Participant follow-up was carried out approximately 14 days after the end of the study to determine if any adverse events had occurred

Participant screening procedures were performed within 28 days prior to study procedures and those who successfully completed these and met the eligibility criteria were invited to check-in to their respective clinical research unit on Day 2. At this point the participants’ eligibility was re-confirmed, then brief (approximately 30 min) training was undertaken on using the *my*blu ENDS device with a 0% nicotine e-liquid to enable the participants to familiarise themselves with use of the device. Baseline (participants using their usual brand of combustible cigarettes) assessments (blood and urine collection) were performed on Days-2 through -1. Subjects were required to abstain from use of any tobacco- or nicotine-containing products for at least 12 h prior to the start of product use on Day 1. A 12 h period was used as this equates to approximately 6 × the half-life of nicotine in human subjects [44] and was deemed a sufficient time period to ensure residual nicotine washout from the subjects’ systems prior to the start of daily procedures in the clinic. This was carried out overnight to prevent impacting on the subjects’ natural product usage behaviour. On Day 1, subjects were randomised to one of eight product use sequences (Fig. 1).

In Part 1 (Days 1–9) of each Study, 20 (intended number) participants were to be randomised to use products A–D and 20 (intended number) subjects randomised to use products E–H from their respective Study (Table 1) (total 80 intended participants in the two Studies combined). On Days 1, 3, 5 and 7, subjects were assigned a *my*blu device and study product for exclusive use for 2 days, according to the randomisation scheme (Fig. 1). On Days 2, 4, 6 and 8, participants took part in controlled product use sessions (i.e., 10 puffs taken at 30 s intervals, 3 s puff duration) for a pharmacokinetic (PK) data not reported in the current study. Following these controlled use sessions, subjects were instructed to continue using their assigned study product ad libitum for the rest of the day until the start of the next overnight abstinence period. On Day 9, participants were given the option to use any or a mixture of the four products they had been assigned (Days 1–8) in an ad libitum manner until 23:00. Urine samples for BoE assessment were collected over 24 h (Days 8–9); blood was collected on Day 9.

Part 2 of the study commenced on the morning of Day 10 and ran to the end of the study on Day 14. The participants recruited to each study (Study 1: *n* = 37; Study 2: *n* = 35) were randomised to one of three study groups (arms): I (Study 1: *n* = 14; Study 2: *n* = 11), J (Study 1: *n* = 11; Study 2: *n* = 10) and K (Study 1: *n* = 12; Study 2: *n* = 12), detailed in Table 2. Note, two participants from Part 1 were not included in the randomisation to Part 2 as they withdrew consent at this point for personal reasons.

**Table 2:** Arms of Part 2 of the study, to which participants were randomised following Part 1

Study arm	Products	Description
I	*my*blu ENDS	Exclusive use of *my*blu ENDS products (A–H) ad libitum
J	Combustible cigarettes	Exclusive smoking of usual brand combustible cigarettes ad libitum
K	Dual use (*my*blu ENDS/combustible cigarette)	Use of usual brand combustible cigarettes up to 50% of the subject’s self-reported use and of *my*blu ENDS products (A–H) ad libitum

Details of products A–H for both Studies 1 and 2 can be found in Table 1

Subjects randomised to exclusive use *my*blu ENDS products (I) and the dual-use arm (K) had the option to use any of the eight study products (A–H) available in Part 1 of their respective Study (Study 1 or 2). Urine samples were collected over 24 h [Days 13 (after the end of Day’s product use) -14]; blood was collected on Day 14. Product use behaviour was assessed by documenting the number of cigarettes smoked, the flavours and strength of the *my*blu ENDS products and pod masses, per day as appropriate (data not shown).

The clinical research units attempted to contact all subjects who used at least one study product (including subjects who terminated the study early), using their standard procedures, approximately 14 days after the last study product use to determine if any adverse events (AEs) had occurred since their last study visit.

### Product use

Use of the assigned products was documented daily and subjects were monitored during clinical confinement to ensure that no illicit nicotine or tobacco products were used. All product use was ad libitum from 07:30 to 23:00 each day apart from on days 2, 4, 6 and 8, on which controlled use sessions were held for additional PK studies (data not shown here). Other exceptions included during meals, overnight abstinences prior to the PK profiling periods, 15 min prior to blood sampling, and 30 min prior to exhaled carbon monoxide (CO) and nitric oxide (NO) measurements. Subjects were not permitted to use any nicotine or tobacco products other than those allocated during the study and were not permitted to remove any study products from their clinic facility.

In the second part of the study, subjects randomised to the dual-use groups were required to request conventional tobacco cigarette products from the clinic staff and smoke only in specified sections of the clinic, away from non-smoking subjects. To standardise cigarette consumption during the study, subjects in the dual-use groups were required to reduce their daily cigarette consumption by ∼50% that reported at screening. Cigarette consumption data (Days 10–14) can be found in Tables 10 and 12 of the supplementary information.

Subjects randomised to receive the *my*blu ENDS products were able to carry them throughout the day, within designated sections of the clinic. New *my*blu ENDS products were supplied to the subjects each morning and throughout the day if the e-liquid solution was fully consumed, if the product failed to work properly, or if the subject requested an alternative flavour (from within their assigned randomisation). All *my*blu ENDS products were weighed before and after use to provide a consumption estimate (Tables 11 and 13, supplementary information).

### Determination of sample size

The selected intended sample size (*n* = 80) was deemed adequate to meet the study objectives, based on previous clinical assessment with *my*blu ENDS products [22]. In a similar BoE assessment using *my*blu products, O’Connell et al. [22] estimated that a sample size of 12 was required to detect a 70% reduction from baseline in participant groups that stopped smoking for the study, and differences between groups could be detected with at least 80% power in two-sided analyses. Their study assigned 15 subjects to each group to maximise the likelihood of a minimum of 12 subjects per group completing the study. Therefore, we intended to allocate the participants of each Study to their respective arms in a ratio of 14:13:13 I:J:K.

### Biomarkers of exposure analyses

The urine and blood BoE measured in this study were selected to represent major classes of HPHCs that have previously been reported for conventional tobacco cigarette smokers [45–50] and are detailed in Table 3. Blood samples for measurement of whole blood COHb were collected in the afternoon of Days-1, 9 and 14. 24 h urine collections were carried out for measurement of NNAL, 3-HPMA, S-PMA, CEMA, HEMA, 3-HMPMA, MHBMA, o-tol, 1-AN, 2-AN, NNN, 1-OHP, 3-OH B[a]P and nicotine equivalents on Days 2–1, 8–9 and 13–14. Each 24 h urine collection commenced at the start of overnight abstinence. Measurement of each biomarker was carried out using validated methods based on: FDA’s Guidance to Industry for Bioanalytical Method Validation [51]; Good Laboratory Practices per 21 CFR Part 58 [52]; and the EMEA Guideline on Bioanalytical Method Validation (EMEA/CHMP/EWP/192217/2009 Rev. 1 Corr.2) [53].

**Table 3:** Biomarkers of exposure (BoE) used in this study and the associated harmful or potentially harmful constituents (HPHC) (present in cigarette smoke)

BoE			HPHC	HPHC category	Associated health risk
Chemical name	Abbreviation	Matrix			
Carboxyhemoglobin	COHb	Blood	Carbon monoxide (CO)	Carbon oxides	RDT
4-(Methylnitrosamino)-1-(3-pyridyl)-1-butanol	NNAL	Urine	4-(Methylnitrosamino)-1-(3-pyridyl)-1-butanone (NNK)	TSNA	CA
3-Hydroxypropyl-mercapturic acid	3-HPMA	Urine	Acrolein	VOC	RT CT
S-Phenyl-mercapturic acid	S-PMA	Urine	Benzene	VOC	CA CT RDT
2-Cyanoethylmercapturic acid	CEMA	Urine	Acrylonitrile	VOC	CA RT
Hydroxyethyl mercapturic acid	HEMA	Urine	Ethylene oxide	VOC	CA RT RDT
3-Hydroxy-1-methylpropyl-mercapturic acid	3-HMPMA	Urine	Crotonaldehyde	VOC	CA
Monohydroxylbutenyl-mercapturic acid	MHBMA	Urine	1,3-Butadiene	VOC	CA RT RDT
o-Toluidine	o-tol	Urine	Toluidine	Aromatic amines	CA
1-Aminonaphthalene	1-AN	Urine	Naphthalene	Aromatic amines	CA RT
2-Aminonaphthalene	2-AN	Urine	Naphthalene	Aromatic amines	CA RT
N-Nitrosonornicotine	NNN	Urine	N-nitrosonornicotine (NNN)	TSNA	CA
1-Hydroxypyrene	1-OHP	Urine	Pyrene	PAH	N/A
3-hydroxybenzo[a]pyrene	3-OH B[a]P	Urine	Benzo[a]pyrene (B[a]P)	PAH	CA
Nicotine equivalents	Nicotine equivalents	Urine	Nicotine	Nicotine	RDT AD

Pyrene is not classified by the FDA but is used as a marker for PAH (including B[a]P) exposure [50]

*TSNA* tobacco specific nitrosamine, *VOC* volatile organic compound, *PAH* polycyclic aromatic hydrocarbon, *RDT* reproductive or developmental toxicant, *CA* carcinogen, *RT* respiratory toxicant, *CT* cardiovascular toxicant, *AD* addictive {as classified by the FDA [37]}

### Data and statistical evaluation

For each urine biomarker, the measured concentration, total biomarker mass excreted per 24 h and creatinine adjusted levels were determined. From these, absolute and percent change from baseline were determined for the mass excreted and creatinine adjusted values. The total urine biomarker mass excreted per 24 h change-from-baseline values were used for the statistical analyses. BoE concentrations reported as below the limit of quantification (BLQ) were reported as such but were set to one half of the limit of quantification for the purpose of data analyses. Statistical Analysis Software (SAS) was used for all data summarisations and statistical analyses. Data were listed by subject, sequence, and study day and summarised by study day (Day 1 and Day 9) in Part 1, by study day (Day 1 and Day 14) in Part 2 for arms I only, and by study arm and study day (Day 9 and Day 14) in Part 2. Absolute and percent change-from-baseline values were also listed and summarised. Descriptive statistics {number of observations [N], mean, median, standard deviation (SD), standard error of the mean (SEM), coefficient of variation (CV; %), minimum, and maximum) were presented; all data summarisations and figures were generated using the Outcomes Population. Using SAS PROC MIXED, comparisons of the Day 9 and Day 1, and Day 14 and Day 1 in the I arms, primary and secondary BoE values were made using a linear mixed model analysis of variance (ANOVA). The ANOVA model included day as a fixed effect and subject as a random effect. Least square (LS) mean values were detailed for each day. LS mean differences, 95% CIs of LS mean differences and *p* values are detailed for the day comparisons. Analyses among the different study arms within the two respective Studies were also carried out as described above, with the arm as the fixed effect and subject as the random effect, and LS means detailed for each arm.

## Results

**Fig. 2:**
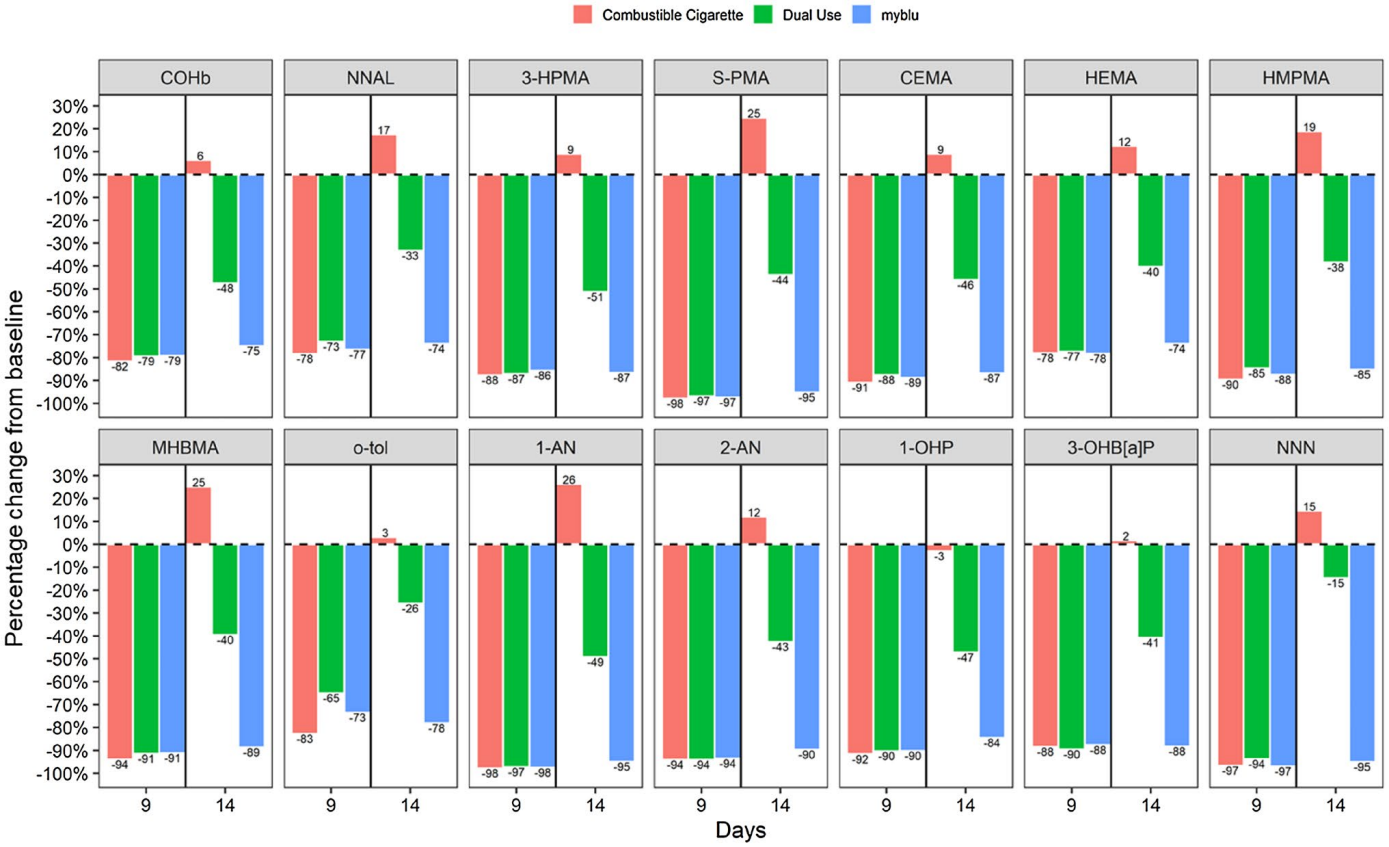
Relative levels of 14 biomarkers of exposure (BoE) following selected product use, measured at Days 9 and 14 of Study 1. Values are expressed relative (%) to baseline levels (combustible cigarette smoking) measured on Day 1 and are detailed in labels on the bars. Red bars represent participants who switched from exclusive *my*blu use to exclusively smoking their usual brand of cigarette on Day 10 of the Study (arm 1 J) (*n* = 11); green bars represent dual-use (50% reported usual brand cigarette smoking and myblu use ad libitum from Day 10 of the Study) participants (arm 1 K) (*n* = 12); blue bars represent participants who continued to exclusively using their selected *my*blu products ad libitum from Day 10 of the Study (arm 1I) (*n* = 14). Definitions of abbreviated BoE can be found in Table 3 (color figure online)

**Fig. 3:**
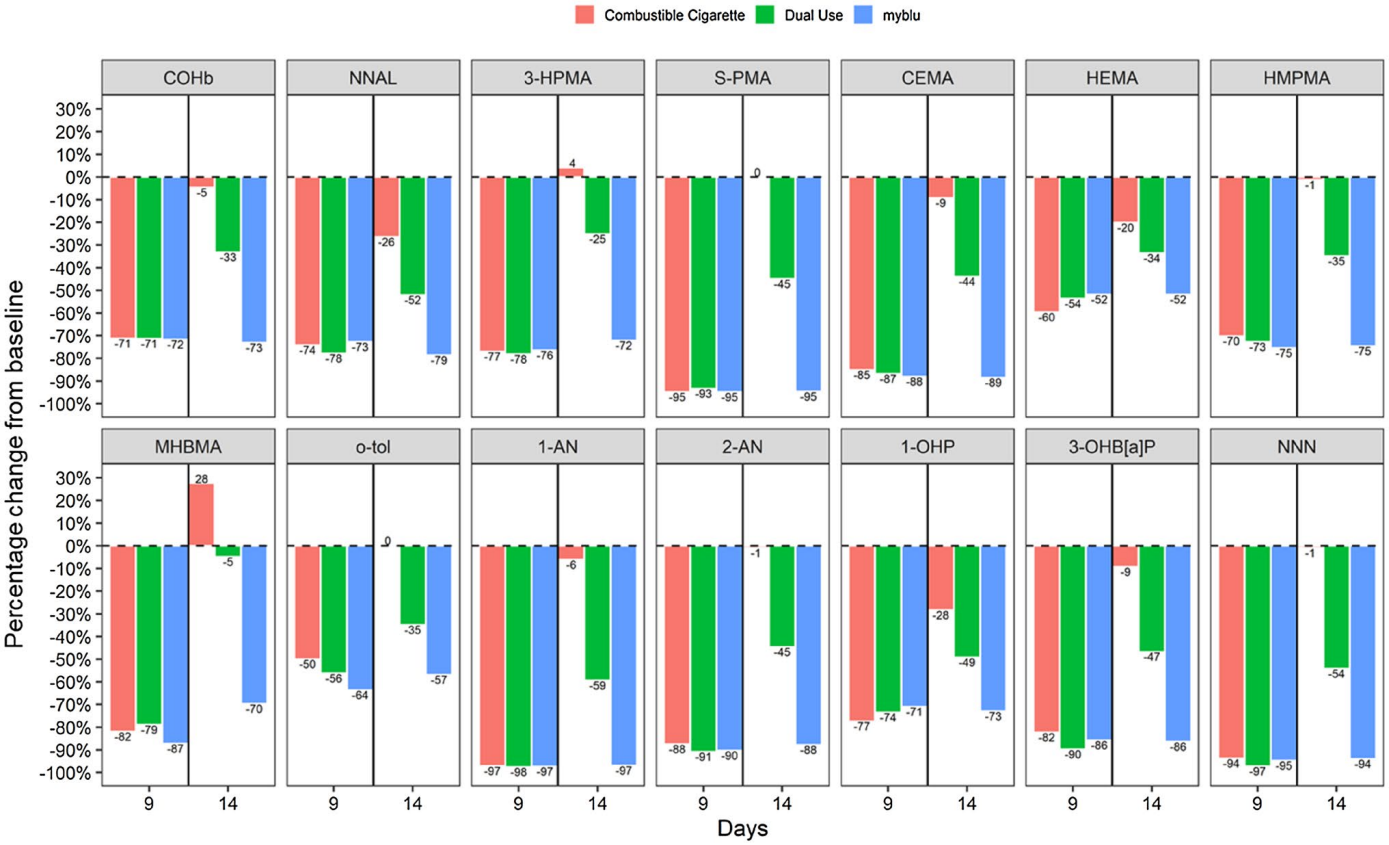
Relative levels of 14 biomarkers of exposure following selected product use, measured at Days 9 and 14 of Study 2. Values are expressed relative (%) to baseline levels (combustible cigarette smoking) measured on Day 1 and are detailed in labels on the bars. Red bars represent participants who switched from exclusive *my*blu use to exclusively smoking their usual brand of cigarette on Day 10 of the Study (arm 2 J) (*n* = 10); green bars represent dual-use (50% reported usual brand cigarette smoking and *my*blu use ad libitum from Day 10 of the Study) participants (arm 2 K) (*n* = 12); blue bars represent participants who continued to exclusively using their selected *my*blu products ad libitum from Day 10 of the Study (arm 2I) (*n* = 11). Definitions of abbreviated BoE can be found in Table 3 (color figure online)

For both Studies 1 and 2, comparisons were made between the measured BoE on Day 1 (baseline; cigarette consumption) and Days 9 or 14 for the exclusive *my*blu use arms (1I and 2I) (Tables 4 and 5, Supplementary information). All BoE, except for nicotine equivalents, demonstrated statistically significant reductions from baseline levels at Days 9 and 14. In Study 1, decreases in the primary BoE (COHb, NNAL, 3-HPMA and S-PMA) from baseline on both Days 9 and 14 ranged from 72 to 97%; in Study 2, this decrease range was 69 to 93%. Similar decreases were also observed for the secondary BoE (mercapturic acid metabolite BoE (CEMA, HEMA, 3-HMPMA, and MHBMA), aromatic amine BoE (o-tol, 1-AN, 2,-AN), 1-OHP, 3-OH B[a]P and NNN), with the range of reduction across Days 9–14 between 70 and 97% for Study 1 and 45 and 97% for Study 2. No statistically significant differences in nicotine equivalent levels compared to baseline were observed in either Study on Days 9 or 14. This was expected as subjects were using their products ad libitum to achieve their preferred level of nicotine satisfaction. Figures 2, 3 detail the levels of 14 BoE (all those measured except nicotine equivalents) at Days 9 and 14 for all the three arms of Studies 1 and 2, respectively, expressed as percentage reduction compared to the relevant baseline levels. More details on the results can be found in the Supplementary information (Tables 4, 5).

Statistical analysis was also carried out between the three product use arms of the respective Studies (Tables 6, 7, Supplementary information). No significant differences were found between the NNN and nicotine equivalent BoE in all three product use arms of Study 1 on comparison of the differences between Days 9 and 14 (part 2 of the study). While no differences were observed between NNAL and HEMA BoE levels in the *my*blu (1I) and dual-use (1 K) arms of Study 1 on comparison of changes between Days 9 and 14, their levels in the combustible cigarette arm (1 J) were significantly different to these two other respective arms. All of the remaining BoE in Study 1 were demonstrated to be significantly different between the three product use arms on comparison of changes between Days 9 and 14. In Study 2, significant differences between the nicotine equivalent BoE across Days 9–14 were observed between the combustible cigarette arm (2 J) and dual-use arm (2 K). However, no differences were observed between the *my*blu arm (2I) and either 2 J or 2 K. On comparison of 2 J and 2 K, there were no significant differences between changes in NNAL and HEMA levels between Days 9 and 14. However, significant differences were observed between 2I and 2 J and between 2I and 2 K for these two BoE. In contrast, no differences in changes between Days 9 and 14 were observed for MHBMA, o-tol and NNN on comparison of 2I and 2 K, whereas differences between 2I and 2 J, and 2 J and 2 K, were statistically significant. All the remaining BoE in Study 2 were found to be significantly different between the three product use arms on comparison of changes between Days 9 and 14.

There were no serious adverse events (SAEs) reported during the product trial. A total of ten mild and one moderate adverse events (AEs) were reported by 11 (14%) subjects. The most frequently reported event was headache, reported by four (10%) subjects. The principal investigators (PIs) considered all events to be unrelated to study product. During Part 1 of the study, a total of 35 mild and three moderate AEs were reported by 27 (34%) subjects across study products. The most frequent event was headache, experienced by six (8%) subjects. All remaining events were reported by four or fewer (≤ 5%) subjects each. The PIs considered two events {throat tightness [*my*blu^™^ Intense Fresh Melon 2.5%] and oropharyngeal discomfort [*my*blu^™^ Gold Leaf 2.4%]} to be likely related to study product and one event {headache [*my*blu^™^ Intense Fresh Mint 2.4%]} to be possibly related. All remaining events were considered to be unlikely related/ unrelated to study product. During Part 2, a total of ten mild and two moderate AEs were reported by nine (11% of total) subjects. The AEs were experienced by four (18%) subjects in the exclusive smoking of usual brand combustible cigarettes group, three (12%) in the exclusive *my*blu^™^ ENDS use group in Study 2, and two (8%) in the Study 1 dual use of *my*blu^™^ ENDS and usual brand combustible cigarettes group. Headache was the most frequently reported event in Part 2, experienced by six (8%) subjects. All remaining events were reported by one (1%) subject each. The PI considered all events in Part 2 unlikely related/unrelated to study product. Full breakdown tables of AEs can be found in the supplementary information (Tables 14–17).

## Discussion

### BoE levels to selected HPHCs were substantially reduced after switching to exclusive *my*blu ENDS use

The first part of this study demonstrated substantial reductions in 14 non-nicotine tobacco smoking-related BoE in adult smokers following 9 days of exclusive use of *my*blu ENDS products across two identical, open label, randomised Studies (1 and 2). Further to this, within the exclusive *my*blu ENDS use arm of Part 2 of the study, 14 days after first switching, these substantial reductions were maintained. Our findings, that switching from combustible cigarette smoking to exclusive fourth generation ENDS use results in significant and rapid reductions in adult smokers’ tobacco smoking-related BoE, are consistent with previous reports, both from clinical settings and at a population level [18, 54–56]. Our studies extend upon previous evidence, for example, we used newer (fourth) generation *my*blu ENDS products than in the study by O’Connell et al. [22, 26]. It has been postulated that newer generation products have been associated with increased BoE of toxicants in users [57], however, our study reported substantial decreases across a comprehensive selection of smoking-related BoEs to HPHCs following exclusive *my*blu ENDS use. Furthermore, the comprehensive nature of the BoE assessed provides a higher degree of confidence that adult smokers who transition to exclusive ENDS use will experience reduced exposure to HPHCs over a short period of time. Our study also assessed a larger number of product variants, from one commercial brand, than previous assessments [17, 22]. These products included a larger selection of flavours, nicotine strengths and two different nicotine formulations, and even when combined within study groups, the reductions in combustible tobacco smoking-associated toxicants can still be demonstrated to be substantial following exclusive *my*blu ENDS use.

The reductions observed in tobacco smoking-related HPHC BoE in this study are perhaps unsurprising as studies on ENDS aerosol chemistry not only provide evidence of reduced HPHC levels, and fewer of these chemicals, in the aerosol itself compared to cigarette smoke [9, 11, 12], but this also correlates with the reduced, or removed, biological effects within toxicological evaluations [8, 38, 58] and in clinical settings [17, 20]. If toxicants are reduced, or even absent, in the ENDS aerosol, user BoE will reflect this directly; this could suggest a re-evaluation on the need for tobacco smoking-related BoE clinical studies for ENDS products where aerosol chemistry studies have fully characterised the aerosol. Our findings add to the weight of evidence that exclusive ENDS use results in the reduction of adult smokers’ exposure to cigarette-associated toxicants and carcinogens and can have an important role in THR.

### Switching to combustible cigarette use reversed reductions in BoE observed after exclusive *my*blu ENDS use

Upon randomised adult smoker switching to exclusive combustible cigarette use in Part 2 of the study, the substantial reductions in BoE measured on Day 9 against baseline were reversed by Day 14. This is consistent with the large numbers of HPHCs found in combustible cigarette smoke [11, 12], to which users will subsequently be exposed. In both Studies, some increases in BoE were observed compared to baseline on Day 14 in the exclusive combustible cigarette smoking participants. Due to the clinical confinement within the study, participants may have increased their product usage compared to baseline due to boredom or lack of usual distractions, as observed in the study by Jay et al. [17]. The reversal of reductions in BoE seen in the participants between Days 9 and 14 indicates that ENDS users will only experience maximal reductions in exposure to harmful tobacco smoking-related compounds upon complete cessation of cigarette smoking. The findings from the dual-use arm of this study reinforce this further.

### Dual use of combustible cigarettes and *my*blu ENDS may have a role in off-ramping from smoking

An important part of THR, through providing alternative, reduced harm, nicotine delivery products, is the off-ramping process of adult combustible cigarette smokers from cigarettes to ENDS, for example. This transitional process typically involves a period in which adult smokers may exercise dual use of combustible cigarettes and ENDS while they familiarise themselves with the new products in terms of usage and formulation preferences. Previous studies have found BoE levels correlated with the level of use of products: Goniewicz et al. [57] documented that higher BoE concentrations in dual-user population samples correlated with greater cigarette smoking frequency and O’Connell et al. [22] found that reductions in BoE levels were generally proportional to the reduction in cigarettes smoked. To our knowledge, there is limited information in the literature on how a period of such dual use of combustible cigarettes and newer generation ENDS affects the BoE levels found in adult smokers. We, therefore, included the dual-use arm in Part 2 of this study to investigate this further. As ENDS use is intended as a substitute, and not a supplement, for cigarette smoking, the arm involved reduction of cigarette use to around 50% of that reported at baseline alongside ad libitum use of the *my*blu ENDS products available.

Following dual use of cigarettes and *my*blu ENDS products, where cigarette smoking is reduced, significant reductions were maintained in the majority of smoking-related HPHC BoE compared to baseline cigarette use. This demonstrates the harm reduction potential of substituting cigarette use with ENDS use, even if only to 50% as a starting point. As expected, these reductions were not to the level of continued exclusive *my*blu use, indicating combustible cigarettes alone lead to increased HPHC exposure. In fact, dual use in Part 2 of the study still brought some BoE levels to statistically similar levels as the exclusive cigarette groups (in Study 1: NNN; in Study 2: NNAL, HEMA). This highlights the importance of complete smoking cessation following a period of dual use, as exclusive *my*blu ENDS use in this study appears to offer the greatest degree of harm reduction potential through the most reduced exposure to tobacco smoking-related BoE. However, and as may be expected, the majority of HPHC BoE levels were recorded to be reduced to around 50% of that seen in the exclusive *my*blu ENDS use arms, indicating reductions in BoE are generally proportional to the reductions in cigarette smoking as observed previously [17, 18, 22, 57]. Due to sample size, only one dual use ratio (50% self-reported daily cigarette consumption plus ad libitum ENDS use) group was included in this study as placing the subjects into smaller groups would reduce the statistical power of the study. However, in the future, it would be interesting to investigate additional patterns of dual usage.

In the dual-use groups, there were some differences observed between the average levels of reduction of specific BoE between Studies 1 and 2, for example, NNN and MHBMA. This may be attributed to the differences in the (majority) nicotine formulation between the two Studies, or may be attributed to individual baseline levels. However this would require further elucidation, perhaps on a product-by-product basis.

### Study products

Of note within this study is the absence of statistical differences between the levels of nicotine equivalents from baseline both at Day 9 and in any of the groups at Day 14. This is expected as users were allowed to use their products ad libitum for the majority of the study, to achieve their desired level of nicotine satisfaction. This also supports the reduced harm potential of ENDS use: preferred nicotine delivery can be achieved in adult smokers, but with substantially reduced levels of several tobacco smoking-related BoE compared to the cigarette smoking baseline.

The selection of products used in this study contained two different nicotine formulations, nicotine salt (nicotine lactate) and freebase nicotine. Nicotine is commonly present in e-liquids in the freebase form, which, due to its higher volatility than nicotine salts, is expected to be generally absorbed in the upper respiratory tract, and reportedly leads to a harsh, bitter sensory experience for the adult smoker [27]. The more recent innovation of using nicotine salts in the e-liquid formulation, which are formed by the reaction of nicotine with a suitable acid, allows improved delivery to, and, therefore, absorption in, the alveoli, and, therefore, increased plasma nicotine levels [22]. This has been shown to provide greater satisfaction for the adult smoker and, therefore, may encourage their use of ENDS as a combustible cigarette smoking alternative [28]. In this study, seven freebase nicotine products and nine nicotine salt products were tested, and these products were generally allocated to Study 1 or 2 dependent on the type of nicotine within their e-liquid formulation. To ensure an even distribution of products between Studies 1 and 2, to allow the product randomisation schedule to be implemented, one nicotine salt product was allocated to Study 2 and was selected for its comparable nicotine strength to the freebase products within that Study. In both Studies 1 and 2, nicotine levels were not significantly changed from their baseline at Days 9 or 14 in the exclusive *my*blu ENDS users. However, nicotine equivalents measured in Study 1 participants (nicotine salt products) were higher compared to in the Study 2 participants (mainly freebase nicotine products), which may be expected given the higher nicotine strengths and greater delivery associated with nicotine salt-containing products [28, 59]. This may also explain the significant difference in nicotine equivalent levels achieved following dual use of combustible cigarettes and the freebase products and exclusive combustible cigarette use. It is also of note, when participants were allowed to use additional products to those they had been allocated in Part 1 of the study, there were slight increases in participants’ nicotine equivalent levels, particularly in the freebase nicotine product users (Study 2). This perhaps reinforces the importance of providing consumers with a range of products, to satisfy their nicotine usage preferences, but could also indicate that participants were becoming more familiar with *my*blu ENDS product usage over the 14 day period of the study.

Due to the large numbers of products used within this study, containing a selection of nicotine strengths, one of two different nicotine formulations and different flavours, it was not possible to analyse formulation-specific BoE. However, the results clearly show, even with a combination of different *my*blu ENDS products, there were significant reductions in the 14 HPHC BoE compared to combustible cigarette baseline levels over a short period of time. The study was designed to allow participants to use a combination of products. This is consistent with the belief that THR can be maximised by providing a broad portfolio of flavours in combination with multiple nicotine strengths to allow adult smokers to find their preferred product(s) as an alternative for smoking combustible cigarettes. A further strength of this aspect of the study design is that it is also reflective of real world exposure to the ENDS category with the many options of combination of different e-liquid formulations. Additionally, the participants were not experienced ENDS users, therefore, it was important to provide them with these choices; in Part 2 of the study participants were also provided with the additional option to choose alternative products to those they had been randomised to in Part 1.

### Study limitations

The present study has a number of limitations and the data should be viewed in this context. While the present study demonstrated reductions on BoE following switching of adult smokers to *my*blu product use, the study did not include an abstinence arm for comparison due to the scale of the wider study. However, similar studies [17, 22] have demonstrated that ENDS use can reduce BoE levels comparable to abstinence itself. The wider study carried out at this time included other analyses including PK and subjective evaluations; these data will be addressed in subsequent publications. If the number of available study arms had permitted, it would have been interesting to compare the results against an appropriate similar product on the market.

The data were not adjusted for multiple comparisons, however, a correction such as Bonferroni could be useful to avoid any false positive outcomes. Application of this correction may have some influence on the outcomes of the study. However, as there were a limited number of groups between which comparisons were being made, this data correction was not included in the objectives of this study. It would be interesting to incorporate this into the analysis of future studies where appropriate.

Upon comparison of the data from the two Studies, baseline BoE levels were generally lower in Study 2 than in Study 1. This is likely due to variability between the sites and also between the subjects on the recruitment databases at each site, e.g., differences in number of combustible cigarette usage per day, however, this was not analysed formally. Additionally, although reductions were observed in NNN levels in Part 2 of the study in the exclusive *my*blu use arm, there was a lack of significance between the arms in some cases (between all arms in Study 1; between I and K in Study 2) which may be attributed to the large variability observed in these values within the subject groups.

Although the study did contain a selection of BoE representing major classes of HPHCs associated with smoking-related disease, the study did not include metals, which have been observed to be present in some e-liquids and are of toxicological interest [60]. Further studies may be required to assess the levels of exposure to metals from ENDS usage. However, the main focus of this study was on the BoE from the FDA HPHC list for tobacco products, on which metals are not listed as hazardous or potentially hazardous constituents [37].

Overall, the data we have presented here adds to the weight of evidence that ENDS can contribute to THR by demonstrating substantial reductions in tobacco smoking-related HPHC BoE in adult smokers following switching to exclusive *my*blu use over a short period of time. Furthermore, dual use of *my*blu ENDS and combustible cigarettes, involving cigarette substitution and representative of a period of off-ramping from tobacco smoking, also leads to reductions in HPHC BoE, but not to the same extent as exclusive ENDS use. The study also demonstrated that the adult smoker participants did not experience any SAEs upon use of any of the *my*blu products, which were deemed to have good safety and tolerability profiles over the 14 days of the study. Future studies, for example into the longer term effects on BoE and tracking biomarkers of potential harm in *my*blu ENDS users, will further inform on the products’ THR potential.

## Supplementary Information

Below is the link to the electronic supplementary material.

Supplementary file1 (DOCX 101 KB)

## Data Availability

Study 1 (CA22749) has been registered at www.clinicaltrials.gov with ID: NCT 04430634. Study 2 (CA22736) study was also registered at www.clinicaltrials.gov with ID: NCT 04429932.

## Abbreviations

AE: Adverse event
1-AN: 1-Aminonaphthalene
2-AN: 2-Aminonaphthalene
ANOVA: Analysis of variance
APPH: Appropriate for the protection of public health
B[a]P: Benzo[a]pyrene
BLQ: Below the limit of quantitation
BoE: Biomarkers of exposure
CEMA: 2-Cyanoethyl-mercapturic acid
CFR: Code of federal regulations
CO: Carbon monoxide
COHb: Carboxyhemoglobin
CI: Confidence interval
ENDS: Electronic nicotine delivery system
FDA: US Food and Drug Administration
GCP: Good clinical practice
GDPR: General data protection regulation
HEMA: Hydroxyethyl mercapturic acid
HPHC: Harmful or potentially harmful constituents
3-HPMA: 3-Hydroxypropylmercapturic acid
3-HMPMA: 3-Hydroxy-1-methylpropylmercapturic acid
ICF: Informed consent form
ICH: International Council for Harmonisation
IRB: Institutional Review Board
LS: Least squares (mean)
MHBMA: Monohydroxybutenylmercapturic acid
NO: Nitric oxide
NNAL: 4-(Methylnitrosamino)-1-(3-pyridyl)-1-butanol
NNK: 4-(Methylnitrosamino)-1-(3-pyridyl)-1-butanone
NNN: N-nitrosonornicotine
3-OH B[a]P: 3-Hydroxybenzo[a]pyrene
1-OHP: Hydroxypyrene
o-tol: O-toluidine
PI: Principal investigator
PK: Pharmacokinetic
SAE: Serious adverse event
S-PMA: S-phenyl mercapturic acid
SD: Standard deviation
SEM: Standard error of the mean
THR: Tobacco harm reduction
