# Supplementary material for: Reductions in biomarkers of exposure to selected harmful and potentially harmful constituents following exclusive and partial switching from combustible cigarettes to myblu™ electronic nicotine delivery systems (ENDS)
Source: Intern Emerg Med. 2021 Aug 26;17(2):397–410. doi: 10.1007/s11739-021-02813-w (PMC8964552; doi:10.1007/s11739-021-02813-w)
Supplement: Supplementary file 1 — Supplementary file1 (DOCX 101 KB) [file 11739_2021_2813_MOESM1_ESM.docx]

**Supplementary Information**

Detailed data, including statistical evaluations for the following article:

***Article title***: Reductions in biomarkers of exposure to selected harmful and potentially harmful constituents, following exclusive and partial switching from combustible cigarettes to *my*blu^TM^ electronic nicotine delivery systems (ENDS)

***Journal***: Internal and Emergency Medicine

***Authors***: Paul Morris^1^, Simon McDermott^2^, Fiona Chapman^2^, Thomas Verron^2^, Xavier Cahours^2^, Matthew Stevenson^2^, Joseph Thompson^2^, Nveed Chaudhary^2, a^, Grant O’Connell^2^

^1^Nerudia Ltd - an Imperial Brands PLC company, Wellington House, Physics Road, Speke, Liverpool, L24 9HP, UK

^2^Imperial Brands PLC, 121 Winterstoke Road, Bristol, BS3 2LL, UK

^a^Broughton Nicotine Services, Oaktree House, W Craven Dr, Earby, Barnoldswick BB18 6JZ

***Corresponding author***: Paul.Morris@nerudia.com


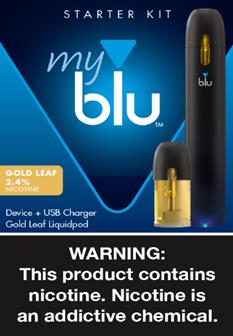


**Figure 4**: Image of a *my*blu ‘starter kit’ containing a device and pod representative of those used in the study.

**Table 4**: Summary of biomarkers of exposure (BoE) levels in Study 1 subjects using *my*blu products exclusively on study Days -1, 9 and 14. Percent (%) changes in BoE between Day -1 and Days 9 and 14 and p-values from comparisons with a linear mixed model analysis of variance are detailed. Significant changes (p<0.05) are denoted by an asterisk (*). The number of subjects included in each evaluation are detailed within brackets ([]). Definitions of abbreviated BoE can be found in **Table 3**.

| **Study 1 BoE** | **Day -1** | **Day 9** | **% Change**  **Day 9 vs Day -1** | **p-value** | **Day 14** | **% Change**  **Day 14 vs Day -1** | **p-value** |
| --- | --- | --- | --- | --- | --- | --- | --- |
| Blood COHb (% Saturation) | 7.69 ± 2.077 [39] | 1.51 ± 0.223 [37] | -79.316 ± 5.6841 [37] | <0.0001* | 1.81 ± 0.690 [14] | -73.162 ± 11.6700 [14] | <0.0001* |
| Urine NNAL (ng/24 hours) | 497.54 ± 257.873 [39] | 127.49 ± 74.680 [37] | -74.270 ± 10.5759 [37] | <0.0001* | 134.96 ± 96.167 [14] | -71.829 ± 13.3843 [14] | <0.0001* |
| Urine 3-HPMA (µg/24 hours) | 1903.26 ± 696.074 [39] | 253.29 ± 105.009 [37] | -85.534 ± 6.7131 [37] | <0.0001* | 261.11 ± 107.359 [14] | -85.918 ± 4.6904 [14] | <0.0001* |
| Urine S-PMA (µg/24 hours) | 8.75 ± 5.573 [39] | 0.19 ± 0.095 [37] | -96.678 ± 3.0057 [37] | <0.0001* | 0.37 ± 0.270 [14] | -94.738 ± 2.6729 [14] | 0.0002* |
| Urine CEMA (µg/24 hours) | 269.78 ± 98.495 [39] | 28.86 ± 9.420 [37] | -88.544 ± 3.5745 [37] | <0.0001* | 34.46 ± 13.052 [14] | -86.275 ± 4.1840 [14] | <0.0001* |
| Urine HEMA (µg/24 hours) | 11.18 ± 8.660 [39] | 2.20 ± 1.254 [37] | -74.067 ± 16.3484 [37] | <0.0001* | 3.31 ± 2.329 [14] | -70.472 ± 17.6998 [14] | 0.0005* |
| Urine 3-HMPMA (µg/24 hours) | 519.36 ± 197.210 [39] | 65.50 ± 20.523 [37] | -85.943 ± 6.4067 [37] | <0.0001* | 73.71 ± 23.094 [14] | -84.356 ± 6.0856 [14] | <0.0001* |
| Urine MHBMA (µg/24 hours) | 5.89 ± 3.989 [39] | 0.35 ± 0.091 [37] | -86.204 ± 19.6285 [37] | <0.0001* | 0.48 ± 0.199 [14] | -84.633 ± 16.1820 [14] | 0.0001* |
| Urine o-tol (ng/24 hours) | 275.87 ± 100.160 [39] | 70.10 ± 22.863 [37] | -71.594 ± 13.0820 [37] | <0.0001* | 60.67 ± 24.837 [14] | -77.052 ± 7.4632 [14] | <0.0001* |
| Urine 1-AN (ng/24 hours) | 181.87 ± 67.990 [39] | 4.76 ± 2.533 [37] | -97.239 ± 1.3759 [37] | <0.0001* | 8.98 ± 3.835 [14] | -94.567 ± 2.0521 [14] | <0.0001* |
| Urine 2-AN (ng/24 hours) | 56.12 ± 20.752 [39] | 3.46 ± 1.296 [37] | -92.868 ± 5.9126 [37] | <0.0001* | 5.50 ± 1.613 [14] | -89.470 ± 2.0700 [14] | <0.0001* |
| Urine NNN (ng/24 hours) | 14.175 ± 11.0190 [39] | 2.937 ± 14.5137 [37] | -82.626 ± 65.4764 [37] | 0.0002* | 0.676 ± 0.4404 [14] | -92.005 ± 9.2182 [14] | 0.0016* |
| Urine 1-OHP (ng/24 hours) | 291.62 ± 123.310 [39] | 28.53 ± 15.120 [37] | -89.484 ± 5.5815 [37] | <0.0001* | 39.51 ± 14.001 [14] | -83.912 ± 4.6141 [14] | <0.0001* |
| Urine 3-OH B[a]P (pg/24 hours) | 230.01 ± 104.013 [39] | 25.74 ± 13.346 [37] | -83.530 ± 25.7603 [37] | <0.0001* | 23.35 ± 10.293 [14] | -87.009 ± 6.0578 [14] | <0.0001* |
| Urine Nicotine Equivalents (mg/24 hours) | 18.74 ± 6.131 [39] | 20.61 ± 11.538 [37] | 13.073 ± 57.1280 [37] | 0.2464 | 21.77 ± 13.927 [14] | 26.626 ± 85.0587 [14] | 0.3996 |

**Table 5**: Summary of biomarkers of exposure (BoE) levels in Study 2 subjects using *my*blu products exclusively on study Days -1, 9 and 14. Percent (%) changes in BoE between Day -1 and Days 9 and 14 and p-values from comparisons with a linear mixed model analysis of variance are detailed. Significant changes (p<0.05) are denoted by an asterisk (*). The number of subjects included in each evaluation are detailed within brackets ([]). Definitions of abbreviated BoE can be found in **Table 3**.

| **Study 2**  **BoE** | **Day -1** | **Day 9** | **% Change**  **Day 9 vs Day -1** | **p-value** | **Day 14** | **% Change**  **Day 14 vs Day -1** | **p-value** |
| --- | --- | --- | --- | --- | --- | --- | --- |
| Blood COHb (% Saturation) | 5.43 ± 1.778 [38] | 1.54 ± 0.280 [35] | -69.336 ± 11.0860 [35] | <0.0001* | 1.50 ± 0.436 [11] | -71.533 ± 9.6820 [11] | <0.0001* |
| Urine NNAL (ng/24 hours) | 287.51 ± 142.944 [38] | 78.15 ± 40.610 [35] | -73.381 ± 9.1682 [35] | <0.0001* | 60.35 ± 38.141 [11] | -75.656 ± 12.4471  11] | 0.0003* |
| Urine 3-HPMA (µg/24 hours) | 1238.91 ± 566.678 [38] | 277.67 ± 79.168 [35] | -74.793 ± 12.1003 [35] | <0.0001* | 344.84 ± 130.342 [11] | -68.726 ± 13.9572 [11] | <0.0001* |
| Urine S-PMA (µg/24 hours) | 4.36 ± 2.906 [38] | 0.22 ± 0.136 [35] | -92.679 ± 6.4276 [35] | <0.0001* | 0.25 ± 0.184 [11] | -92.323 ± 10.3510 [11] | <0.0001* |
| Urine CEMA (µg/24 hours) | 171.89 ± 82.574 [38] | 24.04 ± 11.513 [35] | -85.856 ± 4.8431 [35] | <0.0001* | 25.85 ± 24.193 [11] | -85.435 ± 12.4080 [11] | <0.0001* |
| Urine HEMA (µg/24 hours) | 7.20 ± 6.535 [38] | 2.71 ± 1.263 [35] | -46.317 ± 37.7349 [35] | <0.0001* | 2.33 ± 0.888 [11] | -45.998 ± 28.5653 [11] | 0.0022* |
| Urine 3-HMPMA (µg/24 hours) | 367.29 ± 164.053 [38] | 102.52 ± 37.045 [35] | -69.290 ± 15.5331 [35] | <0.0001* | 93.24 ± 35.451 [11] | -72.148 ± 12.8805 [11] | <0.0001* |
| Urine MHBMA (µg/24 hours) | 2.67 ± 2.007 [38] | 0.33 ± 0.113 [35] | -68.583 ± 44.4135 [35] | <0.0001* | 0.75 ± 0.235 [11] | -60.444 ± 39.8333 [11] | 0.0098* |
| Urine o-tol (ng/24 hours) | 186.17 ± 86.581 [38] | 84.32 ± 40.837 [35] | -50.094 ± 27.7319 [35] | <0.0001* | 96.29 ± 49.516 [11] | -45.164 ± 37.3342 [11] | 0.0005* |
| Urine 1-AN (ng/24 hours) | 153.72 ± 76.266 [38] | 4.52 ± 3.442 [35] | -96.881 ± 2.1383 [35] | <0.0001* | 6.63 ± 10.141 [11] | -95.858 ± 4.7201 [11] | <0.0001* |
| Urine 2-AN (ng/24 hours) | 39.43 ± 17.406 [38] | 4.10 ± 1.598 [35] | -88.501 ± 6.6383 [35] | <0.0001* | 5.35 ± 3.195 [11] | -85.487 ± 8.8000 [11] | <0.0001* |
| Urine NNN (ng/24 hours) | 8.840 ± 6.6919 [38] | 0.484 ± 0.5811 [35] | -93.982 ± 5.6277 [35] | <0.0001* | 0.625 ± 0.7819 [11] | -89.228 ± 12.7057 [11] | 0.0016* |
| Urine 1-OHP (ng/24 hours) | 249.28 ± 119.906 [38] | 73.55 ± 44.815 [35] | -69.018 ± 20.3389 [35] | <0.0001* | 62.17 ± 48.272 [11] | -69.201 ± 18.0465 [11] | 0.0002* |
| Urine 3-OH B[a]P (pg/24 hours) | 178.51 ± 101.133 [38] | 24.05 ± 14.894 [35] | -81.529 ± 20.0152 [35] | <0.0001* | 20.15 ± 20.933 [11] | -79.775 ± 21.1721 [11] | 0.0008* |
| Urine Nicotine Equivalents (mg/24 hours) | 13.30 ± 6.035 [38] | 11.19 ± 8.285 [35] | -16.166 ± 59.6523 [35] | 0.0624 | 16.24 ± 7.173 [11] | 38.984 ± 83.6322 [11] | 0.2491 |

**Table 6**: Statistical comparisons between the BoEs measured in the three arms of Study 1 (I, J, K). The number of participants in each group of the comparison is denoted by n. Least squares (LS) means were calculated for comparison between groups with a linear mixed effect analysis of variance; the 95% confidence interval and p-value are also detailed. Significant differences (p<0.05) are denoted by an asterisk (*). Definitions of chemical name abbreviations can be found in **Table 3**.

| **Study 1 Biomarker** | **Comparison** | **LS Mean**  **Test (n)** | **LS Mean Reference (n)** | **LS Mean Difference (Test - Reference)** | **95% Confidence Interval** | **p-value** |
| --- | --- | --- | --- | --- | --- | --- |
| Blood COHB (% Saturation) | Arm I vs Arm J | 0.35 (14) | 7.48 (11) | -7.13 | -8.41, -5.85 | <0.0001* |
|  | Arm I vs Arm K | 0.35 (14) | 2.91 (12) | -2.56 | -3.81, -1.31 | 0.0002* |
|  | Arm K vs Arm J | 2.91 (12) | 7.48 (11) | -4.57 | -5.90, -3.25 | <0.0001* |
| Urine NNAL (ng/24 hours) | Arm I vs Arm J | 16.23 (14) | 584.59 (11) | -568.36 | -733.66, -403.06 | <0.0001* |
|  | Arm I vs Arm K | 16.23 (14) | 160.73 (12) | -144.50 | -305.90, 16.90 | 0.0777 |
|  | Arm K vs Arm J | 160.73 (12) | 584.59 (11) | -423.86 | -595.11, -252.60 | <0.0001* |
| Urine 3-HPMA (µg/24 hours) | Arm I vs Arm J | -16.45 (14) | 1982.35 (11) | -1998.80 | -2368.47, -1629.14 | <0.0001* |
|  | Arm I vs Arm K | -16.45 (14) | 675.71 (12) | -692.16 | -1053.10, -331.23 | 0.0004* |
|  | Arm K vs Arm J | 675.71 (12) | 1982.35 (11) | -1306.64 | -1689.62, -923.66 | <0.0001* |
| Urine S-PMA (µg/24 hours) | Arm I vs Arm J | 0.19 (14) | 11.82 (11) | -11.63 | -14.69, -8.57 | <0.0001* |
|  | Arm I vs Arm K | 0.19 (14) | 4.84 (12) | -4.65 | -7.64, -1.67 | 0.0032* |
|  | Arm K vs Arm J | 4.84 (12) | 11.82 (11) | -6.98 | -10.14, -3.81 | <0.0001* |
| Urine CEMA (µg/24 hours) | Arm I vs Arm J | 5.20 (14) | 286.49 (11) | -281.29 | -325.91, -236.66 | <0.0001* |
|  | Arm I vs Arm K | 5.20 (14) | 108.39 (12) | -103.19 | -146.76, -59.62 | <0.0001* |
|  | Arm K vs Arm J | 108.39 (12) | 286.49 (11) | -178.09 | -224.32, -131.86 | <0.0001* |
| Urine HEMA (µg/24 hours) | Arm I vs Arm J | 0.64 (14) | 9.76 (11) | -9.12 | -12.24, -6.00 | <0.0001* |
|  | Arm I vs Arm K | 0.64 (14) | 3.33 (12) | -2.69 | -5.74, 0.36 | 0.0816 |
|  | Arm K vs Arm J | 3.33 (12) | 9.76 (11) | -6.43 | -9.66, -3.19 | 0.0003* |
| Urine 3-HMPMA (µg/24 hours) | Arm I vs Arm J | 8.33 (14) | 654.11 (11) | -645.77 | -759.09, -532.45 | <0.0001* |
|  | Arm I vs Arm K | 8.33 (14) | 226.81 (12) | -218.47 | -329.12, -107.83 | 0.0003* |
|  | Arm K vs Arm J | 226.81 (12) | 654.11 (11) | -427.30 | -544.70, -309.90 | <0.0001* |
| Urine MHBMA (µg/24 hours) | Arm I vs Arm J | 0.12 (14) | 8.78 (11) | -8.66 | -11.15, -6.16 | <0.0001* |
|  | Arm I vs Arm K | 0.12 (14) | 3.21 (12) | -3.09 | -5.53, -0.65 | 0.0144* |
|  | Arm K vs Arm J | 3.21 (12) | 8.78 (11) | -5.57 | -8.15, -2.98 | 0.0001* |
| Urine o-tol (ng/24 hours) | Arm I vs Arm J | -10.29 (14) | 284.96 (11) | -295.26 | -348.90, -241.61 | <0.0001* |
|  | Arm I vs Arm K | -10.29 (14) | 88.96 (12) | -99.26 | -151.63, -46.88 | 0.0005* |
|  | Arm K vs Arm J | 88.96 (12) | 284.96 (11) | -196.00 | -251.58, -140.43 | <0.0001* |
| Urine 1-AN (ng/24 hours) | Arm I vs Arm J | 4.65 (14) | 261.98 (11) | -257.33 | -301.81, -212.85 | <0.0001* |
|  | Arm I vs Arm K | 4.65 (14) | 90.30 (12) | -85.65 | -129.07, -42.22 | 0.0003* |
|  | Arm K vs Arm J | 90.30 (12) | 261.98 (11) | -171.68 | -217.76, -125.60 | <0.0001* |
| Urine 2-AN (ng/24 hours) | Arm I vs Arm J | 2.04 (14) | 68.80 (11) | -66.76 | -82.08, -51.45 | <0.0001* |
|  | Arm I vs Arm K | 2.04 (14) | 28.58 (12) | -26.54 | -41.49, -11.58 | 0.0010* |
|  | Arm K vs Arm J | 28.58 (12) | 68.80 (11) | -40.23 | -56.10, -24.36 | <0.0001* |
| Urine NNN (ng/24 hours) | Arm I vs Arm J | 0.217 (14) | 21.478 (11) | -21.261 | -43.98, 1.45 | 0.0657 |
|  | Arm I vs Arm K | 0.217 (14) | 19.528 (12) | -19.312 | -41.49, 2.87 | 0.0858 |
|  | Arm K vs Arm J | 19.528 (12) | 21.478 (11) | -1.950 | -25.48, 21.58 | 0.8673 |
| Urine 1-OHP (ng/24 hours) | Arm I vs Arm J | 12.26 (14) | 315.33 (11) | -303.08 | -371.79, -234.36 | <0.0001* |
|  | Arm I vs Arm K | 12.26 (14) | 112.50 (12) | -100.24 | -167.34, -33.15 | 0.0046* |
|  | Arm K vs Arm J | 112.50 (12) | 315.33 (11) | -202.83 | -274.02, -131.64 | <0.0001* |
| Urine 3-OH B[a]P (pg/24 hours) | Arm I vs Arm J | -0.71 (14) | 270.56 (11) | -271.27 | -361.71, -180.83 | <0.0001* |
|  | Arm I vs Arm K | -0.71 (14) | 115.86 (12) | -116.57 | -204.88, -28.26 | 0.0112* |
|  | Arm K vs Arm J | 115.86 (12) | 270.56 (11) | -154.70 | -248.40, -61.00 | 0.0020* |
| Urine Nicotine Equivalents (mg/24 hours) | Arm I vs Arm J | 2.09 (14) | -0.83 (11) | 2.92 | -3.55, 9.39 | 0.3661 |
|  | Arm I vs Arm K | 2.09 (14) | 5.83 (12) | -3.74 | -10.05, 2.58 | 0.2377 |
|  | Arm K vs Arm J | 5.83 (12) | -0.83 (11) | 6.65 | -0.05, 13.36 | 0.0517 |

**Table 7**: Statistical comparisons between the BoEs measured in the three arms of Study 2 (I, J, K). The number of participants in each group of the comparison is denoted by n. Least squares (LS) means were calculated for comparison between groups with a linear mixed effect analysis of variance; the 95% confidence interval and p-value are also detailed. Significant differences (p<0.05) are denoted by an asterisk (*). Definitions of chemical name abbreviations can be found in **Table 3**.

| **Study 2 Biomarker** | **Comparison** | **LS Mean**  **Test (n)** | **LS Mean Reference (n)** | **LS Mean Difference (Test - Reference)** | **95% Confidence Interval** | **p-value** |
| --- | --- | --- | --- | --- | --- | --- |
| Blood COHB (% Saturation) | Arm I vs Arm J | -0.04 (11) | 3.57 (10) | -3.61 | -4.70, -2.51 | <0.0001* |
|  | Arm I vs Arm K | -0.04 (11) | 2.38 (12) | -2.42 | -3.47, -1.37 | <0.0001* |
|  | Arm K vs Arm J | 2.38 (12) | 3.57 (10) | -1.19 | -2.26, -0.11 | 0.0317* |
| Urine NNAL (ng/24 hours) | Arm I vs Arm J | -13.59 (11) | 137.62 (10) | -151.21 | -206.73, -95.68 | <0.0001* |
|  | Arm I vs Arm K | -13.59 (11) | 87.86 (12) | -101.45 | -154.50, -48.40 | 0.0005* |
|  | Arm K vs Arm J | 87.86 (12) | 137.62 (10) | -49.76 | -104.17, 4.66 | 0.0716 |
| Urine 3-HPMA (µg/24 hours) | Arm I vs Arm J | 57.89 (11) | 1154.20 (10) | -1096.31 | -1480.34, -712.28 | <0.0001* |
|  | Arm I vs Arm K | 57.89 (11) | 625.72 (12) | -567.83 | -934.71, -200.94 | 0.0036* |
|  | Arm K vs Arm J | 625.72 (12) | 1154.20 (10) | -528.48 | -904.81, -152.14 | 0.0075* |
| Urine S-PMA (µg/24 hours) | Arm I vs Arm J | 0.01 (11) | 4.29 (10) | -4.28 | -6.23, -2.33 | 0.0001* |
|  | Arm I vs Arm K | 0.01 (11) | 2.21 (12) | -2.19 | -4.06, -0.33 | 0.0228* |
|  | Arm K vs Arm J | 2.21 (12) | 4.29 (10) | -2.09 | -4.00, -0.17 | 0.0335* |
| Urine CEMA (µg/24 hours) | Arm I vs Arm J | 1.77 (11) | 134.35 (10) | -132.57 | -175.00, -90.15 | <0.0001* |
|  | Arm I vs Arm K | 1.77 (11) | 76.08 (12) | -74.31 | -114.84, -33.78 | 0.0008* |
|  | Arm K vs Arm J | 76.08 (12) | 134.35 (10) | -58.27 | -99.84, -16.69 | 0.0076* |
| Urine HEMA (µg/24 hours) | Arm I vs Arm J | -0.20 (11) | 2.46 (10) | -2.66 | -4.49, -0.82 | 0.0060* |
|  | Arm I vs Arm K | -0.20 (11) | 1.92 (12) | -2.12 | -3.87, -0.37 | 0.0195* |
|  | Arm K vs Arm J | 1.92 (12) | 2.46 (10) | -0.54 | -2.34, 1.26 | 0.5442 |
| Urine 3-HMPMA (µg/24 hours) | Arm I vs Arm J | -2.21 (11) | 282.27 (10) | -284.49 | -374.26, -194.72 | <0.0001* |
|  | Arm I vs Arm K | -2.21 (11) | 119.32 (12) | -121.53 | -207.29, -35.77 | 0.0070* |
|  | Arm K vs Arm J | 119.32 (12) | 282.27 (10) | -162.96 | -250.93, -74.99 | 0.0007* |
| Urine MHBMA (µg/24 hours) | Arm I vs Arm J | 0.41 (11) | 3.22 (10) | -2.81 | -4.36, -1.25 | 0.0009* |
|  | Arm I vs Arm K | 0.41 (11) | 1.46 (12) | -1.05 | -2.54, 0.43 | 0.1586 |
|  | Arm K vs Arm J | 1.46 (12) | 3.22 (10) | -1.75 | -3.28, -0.23 | 0.0257* |
| Urine o-tol (ng/24 hours) | Arm I vs Arm J | 11.97 (11) | 97.59 (10) | -85.62 | -142.42, -28.82 | 0.0044* |
|  | Arm I vs Arm K | 11.97 (11) | 29.65 (12) | -17.68 | -71.95, 36.58 | 0.5109 |
|  | Arm K vs Arm J | 29.65 (12) | 97.59 (10) | -67.94 | -123.60, -12.27 | 0.0184* |
| Urine 1-AN (ng/24 hours) | Arm I vs Arm J | 2.15 (11) | 144.57 (10) | -142.42 | -191.14, -93.69 | <0.0001* |
|  | Arm I vs Arm K | 2.15 (11) | 75.06 (12) | -72.91 | -119.45, -26.36 | 0.0032* |
|  | Arm K vs Arm J | 75.06 (12) | 144.57 (10) | -69.51 | -117.25, -21.76 | 0.0058* |
| Urine 2-AN (ng/24 hours) | Arm I vs Arm J | 1.09 (11) | 35.02 (10) | -33.94 | -45.73, -22.14 | <0.0001* |
|  | Arm I vs Arm K | 1.09 (11) | 19.04 (12) | -17.95 | -29.22, -6.67 | 0.0028* |
|  | Arm K vs Arm J | 19.04 (12) | 35.02 (10) | -15.99 | -27.55, -4.43 | 0.0083* |
| Urine NNN (ng/24 hours) | Arm I vs Arm J | 0.219 (11) | 10.742 (10) | -10.524 | -15.48, -5.57 | 0.0002* |
|  | Arm I vs Arm K | 0.219 (11) | 4.486 (12) | -4.268 | -9.00, 0.47 | 0.0756 |
|  | Arm K vs Arm J | 4.486 (12) | 10.742 (10) | -6.256 | -11.11, -1.40 | 0.0133* |
| Urine 1-OHP (ng/24 hours) | Arm I vs Arm J | -9.87 (11) | 133.30 (10) | -143.17 | -191.46, -94.89 | <0.0001* |
|  | Arm I vs Arm K | -9.87 (11) | 58.56 (12) | -68.43 | -114.56, -22.30 | 0.0050* |
|  | Arm K vs Arm J | 58.56 (12) | 133.30 (10) | -74.74 | -122.06, -27.42 | 0.0030* |
| Urine 3-OH B[a]P (pg/24 hours) | Arm I vs Arm J | 2.59 (11) | 149.75 (10) | -147.16 | -197.49, -96.82 | <0.0001* |
|  | Arm I vs Arm K | 2.59 (11) | 94.25 (12) | -91.66 | -139.75, -43.57 | 0.0005* |
|  | Arm K vs Arm J | 94.25 (12) | 149.75 (10) | -55.50 | -104.83, -6.17 | 0.0287* |
| Urine Nicotine Equivalents (mg/24 hours) | Arm I vs Arm J | 2.58 (11) | -1.33 (10) | 3.91 | -0.28, 8.11 | 0.0666 |
|  | Arm I vs Arm K | 2.58 (11) | 6.05 (12) | -3.46 | -7.48, 0.55 | 0.0880 |
|  | Arm K vs Arm J | 6.05 (12) | -1.33 (10) | 7.38 | 3.26, 11.49 | 0.0010* |

**Table 8**: Study 1 participants’ self reported cigarette use characteristics prior to the study. Data is reported according to study arm allocation. Arm I: exclusive use of myblu products *ad libitum*; J: exclusive smoking of usual brand combustible cigarette *ad libitum*; K: smoking of usual brand combustible cigarettes up to 50% of the subject’s self reported cigarettes per day at the start of the Study and use of *my*blu products *ad libitum*.

|  | | **Study Product Arm** | | |  |
| --- | --- | --- | --- | --- | --- |
| **Trait** |  | **I** | **J** | **K** | **Overall** |
| Brand | Camel | 0 ( 0%) | 1 ( 8%) | 0 ( 0%) | 1 (3%) |
|  | Decade | 0 ( 0%) | 1 ( 8%) | 0 ( 0%) | 1 (3%) |
|  | Eagle 20’s | 0 ( 0%) | 0 ( 0%) | 1 ( 8%) | 1 (3%) |
|  | Edgefield | 1 ( 7%) | 0 ( 0%) | 0 ( 0%) | 1 (3%) |
|  | Kool | 0 ( 0%) | 0 ( 0%) | 1 ( 8%) | 1 (3%) |
|  | L&M | 1 ( 7%) | 2 ( 17%) | 0 ( 0%) | 3 (8%) |
|  | Marlboro | 5 ( 36%) | 7 ( 58%) | 2 ( 17%) | 14 (37%) |
|  | Natural American Spirit | 1 ( 7%) | 0 ( 0%) | 0 ( 0%) | 1 (3%) |
|  | Newport | 2 ( 14%) | 1 ( 8%) | 3 ( 25%) | 6 (16%) |
|  | Pall Mall | 2 ( 14%) | 0 ( 0%) | 1 ( 8%) | 3 (8%) |
|  | Pyramid | 2 ( 14%) | 0 ( 0%) | 2 ( 17%) | 4 (11%) |
|  | USA Gold | 0 ( 0%) | 0 ( 0%) | 1 ( 8%) | 1 (3%) |
|  | Wildhorse | 0 ( 0%) | 0 ( 0%) | 1 ( 8%) | 1 (3%) |
| Flavor | Menthol | 5 ( 36%) | 2 ( 17%) | 4 ( 33%) | 11 (29%) |
|  | Regular | 9 ( 64%) | 10 ( 83%) | 8 ( 67%) | 27 (71%) |
| Length | 100’s | 5 ( 36%) | 5 ( 42%) | 5 ( 42%) | 15 (39%) |
|  | King | 9 ( 64%) | 7 ( 58%) | 7 ( 58%) | 23 (61%) |
| Number of Cigarettes Smoked per Day | 1 1/2 pack (30-39) | 0 ( 0%) | 0 ( 0%) | 1 ( 8%) | 1 (3%) |
|  | 1 pack (20-29) | 4 ( 29%) | 6 ( 50%) | 4 ( 33%) | 14 (37%) |
|  | 10-14 | 3 ( 21%) | 1 ( 8%) | 4 ( 33%) | 8 (21%) |
|  | 15-19 | 6 ( 43%) | 4 ( 33%) | 3 ( 25%) | 13 (34%) |
|  | 2 packs (40+) | 1 ( 7%) | 1 ( 8%) | 0 ( 0%) | 2 (5%) |

**Table 9**: Study 2 participants’ self reported cigarette use characteristics prior to the study. Data is reported according to study arm allocation. Arm I: exclusive use of myblu products *ad libitum*; J: exclusive smoking of usual brand combustible cigarette *ad libitum*; K: smoking of usual brand combustible cigarettes up to 50% of the subject’s self reported cigarettes per day at the start of the Study and use of *my*blu products *ad libitum*.

|  | | **Study Product Arm** | | |  |
| --- | --- | --- | --- | --- | --- |
| **Trait** |  | **I** | **J** | **K** | **Overall** |
| Brand | Camel | 0 (0%) | 0 (0%) | 1 (8%) | 1 (3%) |
|  | Kool | 0 (0%) | 1 (10%) | 0 (0%) | 1 (3%) |
|  | Marlboro | 1 (8%) | 2 (20%) | 2 (15%) | 5 (14%) |
|  | Maverick | 1 (8%) | 1 (10%) | 0 (0%) | 2 (6%) |
|  | Newport | 9 (75%) | 6 (60%) | 10 (77%) | 25 (71%) |
|  | Traffic | 1 (8%) | 0 (0%) | 0 (0%) | 1 (3%) |
| Flavor | Mellow Flavor | 0 (0%) | 1 (10%) | 0 (0%) | 1 (3%) |
|  | Menthol | 11 (92%) | 7 (70%) | 11 (85%) | 29 (83%) |
|  | Non-Menthol | 1 (8%) | 2 (20%) | 2 (15%) | 5 (14%) |
| Cigarette Length | 72 | 2 ( 17%) | 3 ( 30%) | 0 ( 0%) | 5 ( 14%) |
|  | 85 | 2 ( 17%) | 1 ( 10%) | 2 ( 15%) | 5 ( 14%) |
|  | 100 | 8 ( 67%) | 6 ( 60%) | 11 ( 85%) | 25 ( 71%) |
| Number of Cigarettes Smoked per Day | n | 12 | 10 | 13 | 35 |
|  | Mean | 13.3 | 14.4 | 15.7 | 14.5 |
|  | SD | 2.93 | 3.50 | 3.33 | 3.32 |
|  | Minimum | 10 | 10 | 10 | 10 |
|  | Median | 13.5 | 14.5 | 15.0 | 15.0 |
|  | Maximum | 18 | 20 | 20 | 20 |

**Table 10**: Cigarette consumption in arms J (exclusive smoking of usual brand combustible cigarette *ad libitum*) and K (smoking of usual brand combustible cigarettes up to 50% of the subject’s self reported cigarettes per day at the start of the Study and use of *my*blu products *ad libitum*) of Study 1. Data are presented as Mean ± SD [n]. Subject 28 was excluded from Days 10 through 14 because they did not smoke on Days 9 through 13 and most of Day 14.

| **Product** | **Day 10** | **Day 11** | **Day 12** | **Day 13** | **Day 14** |
| --- | --- | --- | --- | --- | --- |
| J | 22.0 ± 5.04 [11] | 23.2 ± 7.15 [11] | 22.5 ± 5.37 [11] | 24.2 ± 6.34 [11] | 23.7 ± 4.56 [11] |
| K | 8.2 ± 2.92 [12] | 8.1 ± 3.50 [12] | 8.7 ± 2.83 [11] | 8.5 ± 2.88 [11] | 8.5 ± 2.84 [11] |

**Table 11**: Average changes in pod weights (g) in Study 1. Arm I: exclusive use of myblu products *ad libitum*; K: smoking of usual brand combustible cigarettes up to 50% of the subject’s self reported cigarettes per day at the start of the Study and use of *my*blu products *ad libitum*. Data are presented as Mean ± SD [n]. Subject 28 was excluded from Days 10 through 14 because they did not smoke on Days 9 through 13 and most of Day 14.

| **Product** | **Day 10** | **Day 11** | **Day 12** | **Day 13** | **Day 14** |
| --- | --- | --- | --- | --- | --- |
| I | 1.82322 ± 0.728527 [14] | 1.97770 ± 0.811665 [14] | 1.75864 ± 0.527212 [14] | 1.89859 ± 0.668284 [14] | 1.89801 ± 0.714312 [14] |
| K | 1.03979 ± 1.080991 [12] | 1.15203 ± 0.921888 [12] | 1.25041 ± 1.343284 [12] | 1.34097 ± 1.257496 [12] | 1.09729 ± 1.010662 [12] |

**Table 12**: Cigarette consumption in arms J (exclusive smoking of usual brand combustible cigarette *ad libitum*) and K (smoking of usual brand combustible cigarettes up to 50% of the subject’s self reported cigarettes per day at the start of the Study and use of *my*blu products *ad libitum*) of Study 2. Data are presented as Mean ± SD [n].

| **Product** | **Day 10** | **Day 11** | **Day 12** | **Day 13** | **Day 14** |
| --- | --- | --- | --- | --- | --- |
| J | 10.9 ± 4.41 [10] | 11.0 ± 3.09 [10] | 11.4 ± 3.47 [10] | 11.3 ± 3.47 [10] | 13.4 ± 3.89 [10] |
| K | 5.9 ± 2.29 [13] | 6.6 ± 1.44 [12] | 7.0 ± 1.91 [12] | 7.1 ± 1.73 [12] | 7.1 ± 1.98 [12] |

**Table 13**: Average changes in pod weights (g) in Study 2. Arm I: exclusive use of myblu products *ad libitum*; J: exclusive smoking of usual brand combustible cigarette *ad libitum;* K: smoking of usual brand combustible cigarettes up to 50% of the subject’s self reported cigarettes per day at the start of the Study and use of *my*blu products *ad libitum*. Data are presented as Mean ± SD [n].

| **Product** | **Day 10** | **Day 11** | **Day 12** | **Day 13** | **Day 14** |
| --- | --- | --- | --- | --- | --- |
| I | 1.28944 ± 0.633386 [12] | 1.60071 ± 1.170587 [12] | 1.69950 ± 0.967297 [12] | 1.40488 ± 0.507572 [12] | 1.29935 ± 0.538456 [11] |
| K | 0.72025 ± 0.409690 [12] | 0.62740 ± 0.562509 [12] | 0.79593 ± 0.627722 [12] | 0.69218 ± 0.584432 [12] | 0.62319 ± 0.479017 [9] |

**Table 14**: Breakdown of adverse events (AEs) reported in Part 1 of Study 1.

|  | | **Product** | | | | | | | | |  |
| --- | --- | --- | --- | --- | --- | --- | --- | --- | --- | --- | --- |
| **Adverse Events*** | **Product Trial** | **A** | **B** | **C** | **D** | **E** | **F** | **G** | **H** | ***my*Blu** | **Overall** |
| Number of Subjects Who Received Study Product | 40 (100%) | 19 (100%) | 19 (100%) | 19 (100%) | 20 (100%) | 19 (100%) | 19 (100%) | 20 (100%) | 20 (100%) | 40 (100%) | 40 (100%) |
| Number of Subjects With Adverse Events | 10 (25%) | 1 (5%) | 4 (21%) | 3 (16%) | 2 (10%) | 3 (16%) | 2 (11%) | 0 (0%) | 3 (15%) | 3 (8%) | 16 (40%) |
| Number of Subjects Without Adverse Events | 30 (75%) | 18 (95%) | 15 (79%) | 16 (84%) | 18 (90%) | 16 (84%) | 17 (89%) | 20 (100%) | 17 (85%) | 37 (93%) | 24 (60%) |
| **Eye disorders** | 0 (0%) | 0 (0%) | 0 (0%) | 0 (0%) | 0 (0%) | 0 (0%) | 0 (0%) | 0 (0%) | 2 (10%) | 0 (0%) | 2 (5%) |
| Abnormal sensation in eye | 0 (0%) | 0 (0%) | 0 (0%) | 0 (0%) | 0 (0%) | 0 (0%) | 0 (0%) | 0 (0%) | 1 (5%) | 0 (0%) | 1 (3%) |
| Eyelid irritation | 0 (0%) | 0 (0%) | 0 (0%) | 0 (0%) | 0 (0%) | 0 (0%) | 0 (0%) | 0 (0%) | 1 (5%) | 0 (0%) | 1 (3%) |
| Eyelid pain | 0 (0%) | 0 (0%) | 0 (0%) | 0 (0%) | 0 (0%) | 0 (0%) | 0 (0%) | 0 (0%) | 1 (5%) | 0 (0%) | 1 (3%) |
| Swelling of eyelid | 0 (0%) | 0 (0%) | 0 (0%) | 0 (0%) | 0 (0%) | 0 (0%) | 0 (0%) | 0 (0%) | 1 (5%) | 0 (0%) | 1 (3%) |
| **Gastrointestinal disorders** | 0 (0%) | 1 (5%) | 1 (5%) | 0 (0%) | 0 (0%) | 0 (0%) | 1 (5%) | 0 (0%) | 0 (0%) | 0 (0%) | 3 (8%) |
| Constipation | 3 (8%) | 1 (5%) | 1 (5%) | 0 (0%) | 0 (0%) | 0 (0%) | 0 (0%) | 0 (0%) | 0 (0%) | 0 (0%) | 2 (5%) |
| Flatulence | 0 (0%) | 0 (0%) | 0 (0%) | 0 (0%) | 0 (0%) | 0 (0%) | 1 (5%) | 0 (0%) | 0 (0%) | 0 (0%) | 1 (3%) |
| **General disorders and administration site conditions** | 0 (0%) | 0 (0%) | 0 (0%) | 0 (0%) | 0 (0%) | 1 (5%) | 0 (0%) | 0 (0%) | 0 (0%) | 0 (0%) | 1 (3%) |
| Drug withdrawal syndrome | 1 (3%) | 0 (0%) | 0 (0%) | 0 (0%) | 0 (0%) | 0 (0%) | 0 (0%) | 0 (0%) | 0 (0%) | 0 (0%) | 0 (0%) |
| Feeling hot | 0 (0%) | 0 (0%) | 0 (0%) | 0 (0%) | 0 (0%) | 1 (5%) | 0 (0%) | 0 (0%) | 0 (0%) | 0 (0%) | 1 (3%) |
| **Investigations** | 0 (0%) | 0 (0%) | 0 (0%) | 0 (0%) | 0 (0%) | 0 (0%) | 0 (0%) | 0 (0%) | 0 (0%) | 1 (3%) | 1 (3%) |
| Blood urine present | 0 (0%) | 0 (0%) | 0 (0%) | 0 (0%) | 0 (0%) | 0 (0%) | 0 (0%) | 0 (0%) | 0 (0%) | 1 (3%) | 1 (3%) |
| **Musculoskeletal and connective tissue disorders** | 0 (0%) | 0 (0%) | 1 (5%) | 1 (5%) | 1 (5%) | 1 (5%) | 0 (0%) | 0 (0%) | 0 (0%) | 0 (0%) | 4 (10%) |
| Back pain | 1 (3%) | 0 (0%) | 1 (5%) | 1 (5%) | 0 (0%) | 1 (5%) | 0 (0%) | 0 (0%) | 0 (0%) | 0 (0%) | 3 (8%) |
| Neck pain | 1 (3%) | 0 (0%) | 0 (0%) | 0 (0%) | 0 (0%) | 0 (0%) | 0 (0%) | 0 (0%) | 0 (0%) | 0 (0%) | 0 (0%) |
| Pain in extremity | 0 (0%) | 0 (0%) | 0 (0%) | 0 (0%) | 1 (5%) | 0 (0%) | 0 (0%) | 0 (0%) | 0 (0%) | 0 (0%) | 1 (3%) |
| **Nervous system disorders** | 0 (0%) | 0 (0%) | 2 (11%) | 1 (5%) | 0 (0%) | 1 (5%) | 1 (5%) | 0 (0%) | 1 (5%) | 2 (5%) | 7 (18%) |
| Dizziness | 0 (0%) | 0 (0%) | 2 (11%) | 0 (0%) | 0 (0%) | 1 (5%) | 1 (5%) | 0 (0%) | 1 (5%) | 0 (0%) | 4 (10%) |
| Headache | 4 (10%) | 0 (0%) | 0 (0%) | 1 (5%) | 0 (0%) | 0 (0%) | 0 (0%) | 0 (0%) | 0 (0%) | 2 (5%) | 3 (8%) |
| **Psychiatric disorders** | 0 (0%) | 0 (0%) | 0 (0%) | 0 (0%) | 1 (5%) | 0 (0%) | 0 (0%) | 0 (0%) | 0 (0%) | 0 (0%) | 1 (3%) |
| Nervousness | 0 (0%) | 0 (0%) | 0 (0%) | 0 (0%) | 1 (5%) | 0 (0%) | 0 (0%) | 0 (0%) | 0 (0%) | 0 (0%) | 1 (3%) |
| **Respiratory, thoracic and mediastinal disorders** | 0 (0%) | 0 (0%) | 0 (0%) | 1 (5%) | 0 (0%) | 0 (0%) | 0 (0%) | 0 (0%) | 0 (0%) | 0 (0%) | 1 (3%) |
| Dysphonia | 0 (0%) | 0 (0%) | 0 (0%) | 1 (5%) | 0 (0%) | 0 (0%) | 0 (0%) | 0 (0%) | 0 (0%) | 0 (0%) | 1 (3%) |
| Throat tightness | 0 (0%) | 0 (0%) | 0 (0%) | 1 (5%) | 0 (0%) | 0 (0%) | 0 (0%) | 0 (0%) | 0 (0%) | 0 (0%) | 1 (3%) |
| **Skin and subcutaneous tissue disorders** | 0 (0%) | 0 (0%) | 1 (5%) | 0 (0%) | 0 (0%) | 1 (5%) | 0 (0%) | 0 (0%) | 0 (0%) | 0 (0%) | 2 (5%) |
| Acne | 0 (0%) | 0 (0%) | 1 (5%) | 0 (0%) | 0 (0%) | 0 (0%) | 0 (0%) | 0 (0%) | 0 (0%) | 0 (0%) | 1 (3%) |
| Blister | 0 (0%) | 0 (0%) | 0 (0%) | 0 (0%) | 0 (0%) | 1 (5%) | 0 (0%) | 0 (0%) | 0 (0%) | 0 (0%) | 1 (3%) |
| Product A: *my*blu™ Intense (nicotine salts), Tobacco flavor, 2.4% Product B: *my*blu™ Intense (nicotine salts), Melon Mint flavor, 3.6% Product C: *my*blu™ Intense (nicotine salts), Fresh Melon flavor, 2.5% Product D: *my*blu™ Intense (nicotine salts), Tangerine Cream flavor, 4.0% Product E: *my*blu™ Intense (nicotine salts), Tobacco flavor, 3.6% Product F: *my*blu™ Intense (nicotine salts), Melon Mint flavor, 2.4% Product G: *my*blu™ Intense (nicotine salts), Fresh Melon flavor, 4.0% Product H: *my*blu™ Intense (nicotine salts), Fresh Mint flavor, 3.6% Subjects used Products A, B, C, D (Group 1) or E, F, G, or H (Group 2) on Days 1 through 8 according to the Part 1 randomization. Subjects could use any product in their assigned group on Day 9 (*my*blu™). Product trial AEs are not included in the overall total. *Adverse events are classified according to MedDRA^®^ Version 22.0. Although a subject may have had 2 or more clinical adverse experiences, the subject is counted only once within a category. The same subject may appear in different categories. | | | | | | | | | | | |

**Table 15**: Breakdown of adverse events (AEs) reported in Part 2 of Study 1.

|  | **Arm** | | |  |
| --- | --- | --- | --- | --- |
| **Adverse Events*** | **I** | **J** | **K** | **Overall** |
| Number of Subjects Who Received Study Product | 14 (100%) | 12 (100%) | 12 (100%) | 38 (100%) |
| Number of Subjects With Adverse Events | 2 (14%) | 2 (17%) | 2 (17%) | 6 (16%) |
| Number of Subjects Without Adverse Events | 12 (86%) | 10 (83%) | 10 (83%) | 32 (84%) |
| **Eye disorders** | 1 (7%) | 0 (0%) | 0 (0%) | 1 (3%) |
| Ocular hyperaemia | 1 (7%) | 0 (0%) | 0 (0%) | 1 (3%) |
| **Gastrointestinal disorders** | 1 (7%) | 0 (0%) | 0 (0%) | 1 (3%) |
| Toothache | 1 (7%) | 0 (0%) | 0 (0%) | 1 (3%) |
| **General disorders and administration site conditions** | 0 (0%) | 1 (8%) | 0 (0%) | 1 (3%) |
| Chest pain | 0 (0%) | 1 (8%) | 0 (0%) | 1 (3%) |
| **Musculoskeletal and connective tissue disorders** | 0 (0%) | 0 (0%) | 1 (8%) | 1 (3%) |
| Pain in extremity | 0 (0%) | 0 (0%) | 1 (8%) | 1 (3%) |
| **Nervous system disorders** | 1 (7%) | 1 (8%) | 1 (8%) | 3 (8%) |
| Headache | 1 (7%) | 1 (8%) | 1 (8%) | 3 (8%) |
| **Skin and subcutaneous tissue disorders** | 1 (7%) | 0 (0%) | 0 (0%) | 1 (3%) |
| Rash papular | 1 (7%) | 0 (0%) | 0 (0%) | 1 (3%) |
| Arm I: Exclusive use of *my*blu™ products ad libitum Arm J: Exclusive smoking of usual brand combustible cigarettes ad libitum Arm K: Smoking of usual brand combustible cigarettes (up to 50% of the subject’s self-reported CPD) and use of *my*blu™ products ad libitum *Adverse events are classified according to MedDRA^®^ Version 22.1 Although a subject may have had 2 or more clinical adverse experiences, the subject is counted only once within a category. The same subject may appear in different categories. | | | | |

**Table 16**: Breakdown of adverse events (AEs) reported in Part 1 of Study 2.

|  | | **Product** | | | | | | | | |  |
| --- | --- | --- | --- | --- | --- | --- | --- | --- | --- | --- | --- |
| **Adverse Events*** | **Product Trial** | **A** | **B** | **C** | **D** | **E** | **F** | **G** | **H** | ***my*blu™** | **Overall** |
| Number of Subjects Who Received Study Product | 39 (100%) | 18 (100%) | 16 (100%) | 16 (100%) | 18 (100%) | 20 (100%) | 20 (100%) | 20 (100%) | 20 (100%) | 35 (100%) | 39 (100%) |
| Number of Subjects With Adverse Events | 1 (3%) | 3 (17%) | 1 (6%) | 1 (6%) | 1 (6%) | 2 (10%) | 1 (5%) | 0 (0%) | 1 (5%) | 2 (6%) | 11 (28%) |
| Number of Subjects Without Adverse Events | 38 (97%) | 15 (83%) | 15 (94%) | 15 (94%) | 17 (94%) | 18 (90%) | 19 (95%) | 20 (100%) | 19 (95%) | 33 (94%) | 28 (72%) |
| **Gastrointestinal disorders** | 0 (0%) | 0 (0%) | 1 (6%) | 0 (0%) | 0 (0%) | 0 (0%) | 1 (5%) | 0 (0%) | 0 (0%) | 0 (0%) | 2 (5%) |
| Constipation | 0 (0%) | 0 (0%) | 1 (6%) | 0 (0%) | 0 (0%) | 0 (0%) | 0 (0%) | 0 (0%) | 0 (0%) | 0 (0%) | 1 (3%) |
| Diarrhoea | 0 (0%) | 0 (0%) | 0 (0%) | 0 (0%) | 0 (0%) | 0 (0%) | 1 (5%) | 0 (0%) | 0 (0%) | 0 (0%) | 1 (3%) |
| **Infections and infestations** | 0 (0%) | 0 (0%) | 0 (0%) | 0 (0%) | 0 (0%) | 0 (0%) | 0 (0%) | 0 (0%) | 0 (0%) | 1 (3%) | 1 (3%) |
| Upper respiratory tract infection | 0 (0%) | 0 (0%) | 0 (0%) | 0 (0%) | 0 (0%) | 0 (0%) | 0 (0%) | 0 (0%) | 0 (0%) | 1 (3%) | 1 (3%) |
| **Nervous system disorders** | 0 (0%) | 2 (11%) | 0 (0%) | 0 (0%) | 1 (6%) | 0 (0%) | 0 (0%) | 0 (0%) | 1 (5%) | 1 (3%) | 5 (13%) |
| Headache | 0 (0%) | 1 (6%) | 0 (0%) | 0 (0%) | 0 (0%) | 0 (0%) | 0 (0%) | 0 (0%) | 1 (5%) | 1 (3%) | 3 (8%) |
| Hypoaesthesia | 0 (0%) | 1 (6%) | 0 (0%) | 0 (0%) | 0 (0%) | 0 (0%) | 0 (0%) | 0 (0%) | 0 (0%) | 0 (0%) | 1 (3%) |
| Migraine | 0 (0%) | 0 (0%) | 0 (0%) | 0 (0%) | 1 (6%) | 0 (0%) | 0 (0%) | 0 (0%) | 0 (0%) | 0 (0%) | 1 (3%) |
| **Reproductive system and breast disorders** | 0 (0%) | 0 (0%) | 0 (0%) | 0 (0%) | 0 (0%) | 2 (10%) | 0 (0%) | 0 (0%) | 0 (0%) | 0 (0%) | 2 (5%) |
| Dysmenorrhoea | 0 (0%) | 0 (0%) | 0 (0%) | 0 (0%) | 0 (0%) | 2 (10%) | 0 (0%) | 0 (0%) | 0 (0%) | 0 (0%) | 2 (5%) |
| **Respiratory, thoracic and mediastinal disorders** | 0 (0%) | 1 (6%) | 0 (0%) | 0 (0%) | 0 (0%) | 0 (0%) | 0 (0%) | 0 (0%) | 0 (0%) | 0 (0%) | 1 (3%) |
| Oropharyngeal discomfort | 0 (0%) | 1 (6%) | 0 (0%) | 0 (0%) | 0 (0%) | 0 (0%) | 0 (0%) | 0 (0%) | 0 (0%) | 0 (0%) | 1 (3%) |
| **Skin and subcutaneous tissue disorders** | 0 (0%) | 0 (0%) | 0 (0%) | 1 (6%) | 0 (0%) | 0 (0%) | 0 (0%) | 0 (0%) | 0 (0%) | 0 (0%) | 1 (3%) |
| Dermatitis contact | 0 (0%) | 0 (0%) | 0 (0%) | 1 (6%) | 0 (0%) | 0 (0%) | 0 (0%) | 0 (0%) | 0 (0%) | 0 (0%) | 1 (3%) |
| **Vascular disorders** | 1 (3%) | 0 (0%) | 0 (0%) | 0 (0%) | 0 (0%) | 0 (0%) | 0 (0%) | 0 (0%) | 0 (0%) | 0 (0%) | 0 (0%) |
| Hypertension | 1 (3%) | 0 (0%) | 0 (0%) | 0 (0%) | 0 (0%) | 0 (0%) | 0 (0%) | 0 (0%) | 0 (0%) | 0 (0%) | 0 (0%) |
| Product A: *my*blu™ (freebase), Gold Leaf flavor, 2.4% Product B: *my*blu™ (freebase), Polar Mint flavor, 2.4% Product C: *my*blu™ (freebase), Cherry flavor, 2.4% Product D: *my*blu™ (freebase), Vanilla flavor, 2.4% Product E: *my*blu™ (freebase), Gold Leaf flavor, 1.2% Product F: *my*blu™ (freebase), Polar Mint flavor, 1.2% Product G: *my*blu™ (freebase), Menthol flavor, 2.4% Product H: *my*blu™ Intense (nicotine salts), Fresh Mint flavor, 2.4% Subjects used Products A, B, C, D (Group 1) or E, F, G, or H (Group 2) on Days 1 through 8 according to the Part 1 randomization. Subjects could use any product in their assigned group on Day 9 (*my*blu™). Product trial AEs are not included in the overall total. *Adverse events are classified according to MedDRA^®^ Version 22.0. Although a subject may have had 2 or more clinical adverse experiences, the subject is counted only once within a category. The same subject may appear in different categories. | | | | | | | | | | | |

**Table 17**: Breakdown of adverse events (AEs) reported in Part 2 of Study 2.

|  | **Arm** | | |  |
| --- | --- | --- | --- | --- |
| **Adverse Events*** | **I** | **J** | **K** | **Overall** |
| Number of Subjects Who Received Study Product | 12 (100%) | 10 (100%) | 13 (100%) | 35 (100%) |
| Number of Subjects With Adverse Events | 1 (8%) | 2 (20%) | 0 (0%) | 3 (9%) |
| Number of Subjects Without Adverse Events | 11 (92%) | 8 (80%) | 13 (100%) | 32 (91%) |
| **Nervous system disorders** | 1 (8%) | 2 (20%) | 0 (0%) | 3 (9%) |
| Headache | 1 (8%) | 2 (20%) | 0 (0%) | 3 (9%) |
| Arm I: Exclusive use of *my*blu™ products *ad libitum* Arm J: Exclusive smoking of usual brand combustible cigarettes *ad libitum* Arm K: Smoking of usual brand combustible cigarettes (up to 50% of the subject’s self-reported CPD) and use of *my*blu™ products *ad libitum* *Adverse events are classified according to MedDRA^®^ Version 22.0. Although a subject may have had 2 or more clinical adverse experiences, the subject is counted only once within a category. The same subject may appear in different categories. | | | | |
